# Seascape connectivity: evidence, knowledge gaps and implications for temperate coastal ecosystem restoration practice and policy

**DOI:** 10.1038/s44183-025-00128-3

**Published:** 2025-06-12

**Authors:** J. Preston, A. Debney, C. Gamble, M. J. Hardy, G. J. C. Underwood, A. Garbutt, J. Harley, R. Baker, R. M. Dunk, M. Grigg, B. T. Hancock, I. W. Hendy, E. C. La Marca, J. Murray, N. Pettorelli, S. J. Pittman, S. E. Reeves, M. Robertson, A. M. Sturrock, R. H. Thurstan, R. K. F. Unsworth, E. A. Ward, S. L. Ward, G. J. Watson, S. C. L. Watson, L. M. Wedding, T. A. Worthington, R. A. Wright, C. Yesson, P. S. E. zu Ermgassen

**Affiliations:** 1 Institute of Marine Sciences, School of the Environment and Life Sciences, University of Portsmouth, Portsmouth, PO4 9LY UK; 2 Zoological Society of London (ZSL), Regent’s Park, London, NW1 4RY UK; 3 School of Life Sciences, University of Essex, Colchester, Essex CO4 3SQ UK; 4 UK Centre for Ecology and Hydrology, Environment Centre Wales, Bangor, LL57 2UW UK; 5 Stokes School of Marine and Environmental Sciences, University of South Alabama, Dauphin Island Sea Lab, Dauphin Island, AL USA; 6 Department of Natural Sciences, Manchester Metropolitan University, Manchester, M1 5GD UK; 7 The Nature Conservancy, URI Graduate School of Oceanography, Narragansett, RI 02882 USA; 8 Institute of Anthropic Impacts and Sustainability in Marine Environment, National Research Council (IAS-CNR), Lungomare Cristoforo Colombo 4521, Loc. Addaura, 90149 Palermo, Italy; 9 Blue Marine Foundation, South Building, Somerset House, London, WC2R 1LA UK; 10 Oxford Seascape Ecology Lab, School of Geography and the Environment, University of Oxford, Oxford, OX1 3QY UK; 11 Seascape Analytics Ltd, Plymouth, UK; 12 The Nature Conservancy Australia, Carlton, VIC Australia; 13 Esmée Fairbairn Foundation, London, UK; 14 Centre for Ecology and Conservation, University of Exeter, Cornwall, TR10 9FE UK; 15 Biosciences, Faculty of Science and Engineering, Swansea University, Swansea, Wales UK; 16 Project Seagrass, Unit 1, Garth Drive, Brackla Industrial Estate, Bridgend, Wales UK; 17 School of Ocean Sciences, Bangor University, Menai Bridge, Isle of Anglesey, Wales UK; 18 Plymouth Marine Laboratory, Prospect Place, Plymouth, Devon, PL13DH UK; 19 Conservation Science Group, Department of Zoology, University of Cambridge, Cambridge, CB2 3EJ UK; 20 Changing Oceans Group, School of Geosciences, University of Edinburgh, Edinburgh, EH9 3FE UK

**Keywords:** Restoration ecology, Marine biology

## Abstract

Temperate coastal marine ecosystems have undergone severe global loss and degradation. We provide a framework for considering ecological connectivity in marine systems and evidence for ecological connectivity across temperate coastal seascapes, developed through expert consensus and structured review. We demonstrate that ecosystem functioning and the delivery of ecosystem services require the existence of a healthy mosaic of coastal habitats, maintained by the exchanges of matter and energy between them. We advocate a seascape approach, that restores connectivity and optimal structure-function relationships, is crucial for successful ecosystem restoration. Consequently, we provide recommendations to deliver seascape restoration of coastal habitats to support the targets set by the 2021-30 UN Decades of Ocean Science and Ecosystem Restoration. Acknowledging the interconnected nature of coastal ecosystems has implications for policy. We identify opportunities and actions to support nature recovery and integrate policy frameworks across climate and biodiversity agendas to achieve international goals for planetary resilience.

## Introduction

The triple planetary crises of global climate change, biodiversity loss and pollution^1^ that humanity has both caused and now faces, are interlinked phenomena that are played out acutely at the land-sea interface^2–5^. While coasts are areas of high productivity^6^, biodiversity and natural resources^7^, they are also inhabited by 37% of the global population^8,9^ and heavily impacted by pollution^10^ and habitat loss^11^. Anthropogenic stressors have diminished nearly all coastal marine habitats, and globally 87% of marine biomes are impacted by overfishing, pollution and climate change^12^. In temperate regions, biogenic habitats have been decimated over the last 200 years due to destructive fishing methods, land use changes and eutrophication linked to coastal development^13–15^, as well as stressors including introduced species, disease and pollution^16,17^. The cumulative effects of these impacts have created a severely shifted baseline, and the reduced and fragmented range of extant biogenic habitats across the temperate coastal seascapes will have implications from the perspective of ecological connectivity.

The persistence of anthropogenic impacts, coupled with the lack of scientific records of previous states, means that long-term changes are often difficult to quantify and are readily overlooked without the aid of historical ecological studies^18–20^. Marine biodiversity losses have been accelerating since 1800 AD, with an estimated average >50% decline in abundance for 91% of assessed species, at the global scale^15,21^. Saltmarsh habitats are highly vulnerable to land reclamation for agriculture and development, and 50% of global saltmarshes are lost or degraded^22^, with higher regional examples; >50% of European saltmarsh habitats^13^ and 90% of saltmarsh habitats in the United Kingdom (UK)^23,24^ have been lost. This is coupled with the loss of interconnecting tidal flats^25^. The rate of seagrass loss accelerated globally from pre-1940 to 1990^26^, with unknown associated declines in biodiversity, although this has been quantified for some regions (e.g., UK; 44-90% habitat loss with associated biomass loss of 400 million fish^27^). Kelp forests are in decline across all continents at an annual rate of 1.8% loss globally, and of those remaining, between 40–60% are degraded^28^. Globally, biogenic oyster reefs are considered to have declined by 85%^29^ and *Ostrea edulis* reefs are classified as a collapsed ecosystem in Europe^30^ when assessed according to the IUCN Red List of Ecosystem criteria. The evidence is clear, the connections across habitats and trophic webs of the temperate coastal seascape have been disrupted, weakened or severed by anthropogenic activity; leaving remnant and incomplete trophic webs in place, and in some cases leading to trophic collapse and ecological phase shifts to alternative degraded ecosystems^31–33^. For habitats that persist, transformations resulting from climate-driven changes are increasingly observed. For example, rapid ocean warming in temperate Australia and elsewhere has driven the replacement of temperate kelp forests by seaweed turfs and species characteristic of sub-tropical communities^34,35^, while saltmarsh and other coastal wetlands are at extreme risk globally from erosion and inundation due to sea-level rise^36,37^.

Nature based Solutions (NbS), are recognised by the United Nations (UN) as actions that protect and restore ecosystems; address social, economic or environmental challenges, *whilst* simultaneously delivering human wellbeing through ecosystem services, resilience and biodiversity benefits (2022 UNEP/EA.5/Res.5). An increasing evidence base illustrates the critical value of coastal habitats individually for climate regulation through carbon storage (e.g., seagrass meadows^38^, saltmarsh^39^), supporting water quality via nutrient cycling (e.g., oyster reefs^40,41^) the provisioning of fishery production via nursery function^42,43^, and protection via coastal defences^44^. It is these ecosystem functions and services that NbS depend on to deliver climate mitigation and adaptation, remediate pollution, reverse biodiversity loss and ensure the flow of benefits to people^45^. However, the mechanisms and evidence for ecological connectivity across coastal habitats, and its role in modulating ecosystem service delivery in temperate coastal systems, has yet to be explored in detail. Such understanding is of relevance to delivering global targets including the Paris Agreement, Sustainable Development Goals (SDGs) and Kunming-Montreal Global Biodiversity Framework (GBF), particularly Target 2 that requires actions to ‘*Ensure that by 2030 at least 30 per cent of areas of degraded terrestrial, inland water, and coastal and marine ecosystems are under effective restoration, in order to enhance biodiversity and ecosystem functions and services, ecological integrity and connectivity*’.

Momentum is now building to address the triple planetary crises through NbS, with the Global Biodiversity Framework and the current UN Decades (2021–2030) of both Ecosystem Restoration and Ocean Science for Sustainable Development (the Ocean Decade) setting ambitious targets to revive ocean ecosystems^46–48^. Key outcomes of the Ocean Decade include ‘*a healthy and resilient ocean*’ and ‘*an accessible ocean*’, which are to be achieved by enhancing the science-policy interface and mapping marine ecosystems and habitats, pressures and ecosystem services at scale to enable best practice management^49^. The compelling case for biodiversity conservation, provided by the Dasgupta Review on the Economics of Biodiversity, also highlighted the need for integrated policies that understand ecosystem complexity and the multiple values of nature^50^. The IPCC Sixth Assessment Report (Climate Change 2022: Impacts, Adaptation and Vulnerability) similarly called for urgent cooperative action to protect and restore ecosystems to safeguard biodiversity and associated climate resilience, particularly within coastal systems^51^. Progressing towards these outcomes and Sustainable Development Goal 14 (the sustainable use and management of oceans and marine resources) will require a transformative shift to pooling knowledge on ecosystems and the NbS they provide, to capitalise on the many potential benefits of approaching restoration at the seascape scale^52^.

There are growing global and national habitat-specific networks for saltmarshes, seagrass meadows, kelp forests and native oyster reefs that include scientists, regulators and practitioners to apply ecological expertise to the conservation, recovery or restoration of these habitats (e.g., Society for Ecological Restoration (SER), European Native Oyster Restoration Alliance (NORA), Australian Seagrass Restoration Network, Kelp Forest Alliance). Whilst the development of effective and scalable habitat specific approaches remains vitally important, there is also an ideological shift towards a more integrated and multi-habitat approach to the understanding of landscapes and seascapes as socio-ecological systems which is influencing restoration theory and practice^53–55^ and better aligns with international policy targets (e.g, Article 2 of the Convention on Biological Diversity).

Connectivity has long been recognised as key to biodiversity dynamics in terrestrial environments, with landscape ecology having provided a well-developed conceptual and operational framework for addressing complex multi-scale questions regarding the influence of spatial patterning on ecological processes^56^. Landscape ecology, therefore, provides an important foundational perspective in conceptualising the dynamics of spatially heterogeneous areas by explicitly focusing on the linkages between spatial patterns and ecological processes^57^. As a result, landscape ecology has become highly relevant to solution-oriented disciplines such as sustainability science, as evidenced by its application to support decision making in terrestrial conservation and restoration planning^58–60^.

Like landscape ecology, connectivity is a central theme of seascape ecology^61,62^, broadly defined as *the study of the causes and ecological consequences of spatial and temporal patterning on marine systems*^63–65^. A seascape ecology approach recognises that oceans and coasts are spatially heterogeneous systems exhibiting complex dynamics and interconnections across multiple spatial and temporal scales^62^, and this complexity can be explored through a pattern-oriented set of concepts and tools familiar to landscape ecologists^66^. Principles and methodologies developed in landscape and seascape ecology have contributed to the development of ecosystem-based management with its central focus on the linkages between ecosystem structure and function and the importance of considering multiple scales of interconnectivity among the system components.

Although coastal habitats are frequently classified into distinct habitat types (e.g., maerl bed, seagrass, saltmarsh, etc.), they exist as part of a wider mosaic of interconnected habitat patches^67,68^. Habitat heterogeneity, biogenic habitats, ecosystem engineers and keystone species provide the framework for functioning coastal food webs, and connectivity between these habitats and ecosystems is the mechanism by which these trophic systems are supported and maintained^68,69^. For instance, many species occupying coastal ecosystems connect different habitat patches with their daily, seasonal and ontogenetic movements^42,70^. Fish ecologists have documented many examples of juvenile fish requiring structured nearshore habitats such as seagrass, saltmarsh or shellfish reef for survival and growth, before shifting their home range farther offshore^43,71^.

Connectivity, highlighted as a critical factor for the success of nature recovery strategies in terrestrial systems^72^, is also recognised as essential for delivery and quality of ecosystem services from marine restoration^73^. Connectivity enables energy, nutrient and genetic material transfers between ecosystems; it shapes access to key habitats for many species (e.g., nursery grounds), directly impacting their behaviour, growth, survival, and spatial distributions^68,74^. Enhanced ecological connectivity can increase functionality across interacting terrestrial and marine habitats that span the land-sea continuum^72^. For example, it was recently demonstrated that natural and restored oyster reefs situated adjacent to saltmarsh and seagrass habitats had higher fish and invertebrate densities^75^. These findings suggest that adopting a multi-habitat and multi-trophic restoration approach that considers seascape ecological connectivity at its core could yield similar positive outcomes. This relatively recent scientific perspective reflects much older Traditional Ecological Knowledge (TEK), held by indigenous peoples, and of relevance to marine restoration^76,77^. Marine restoration could therefore benefit from considering these TEK perspectives alongside our evolving scientific understanding, summarised in the 5Cs of Seascape Ecology: Context, Configuration, Connectivity, Consideration of scale and Culture^62,78,79^.

This paper presents a framework for marine ecological connectivity, reviews the evidence for ecological connectivity between major biogenic and vegetated coastal habitats across the temperate seascape (Table 1), assesses the role of connectivity in ecosystem functioning and the delivery of ecosystem services, and considers the implications of this understanding of ecological connectivity for the policy and practice of restoring coastal ecosystems. Expert opinion gathered at a symposium on ‘Ecological connectivity across temperate coastal habitats’ held at the Zoological Society of London (ZSL), combined with a structured review, has been used to develop the concepts and evidence of ecological connectivity and highlight key knowledge gaps. This is framed around the themes of biodiversity, climate mitigation and adaptation, and bioremediation to highlight the essential role that a healthy temperate coastal seascape can play in mitigating the triple planetary crisis.

**Table 1:** Major biogenic and vegetated habitats occurring across the temperate coastal seascape, from upper intertidal to subtidal zones: Vertical zonation, habitat description and global status are supported by a reference list (Supplementary References 1)




*some species within habitats may extend outside of these ranges.

Zonation codes refer to *SL* Supralittoral and littoral fringe, *UE* Upper Eulittoral, *ME* Mid Eulittoral, *LE* Lower Eulittoral, *SI* Sublittoral & Infralittoral (0-20m), *CL* Circalittoral (-20 to -80m)*. Depths for subtidal zones given in relation to Chart Datum.

Considering the implications of this evidence, we define and conceptualise a seascape approach to restoration as necessary to build ecological resilience, restore habitat heterogeneity and trophic complexity that underpins biodiversity, and to provide the natural processes that deliver the ecosystem services we urgently require to address the triple planetary crisis^22^. We provide policy pathways and practical recommendations to achieve seascape restoration of coastal habitats – at local to global scales – that account for connectivity and how it underpins the delivery of NbS if we are to reach targets outlined in initiatives like the UN Decades, Paris Agreement and GBF and multinational targets proposed in the EU Nature Restoration Law^80^.

## Results

### Defining the seascape concept and approach to restoration

**Fig. 1:**
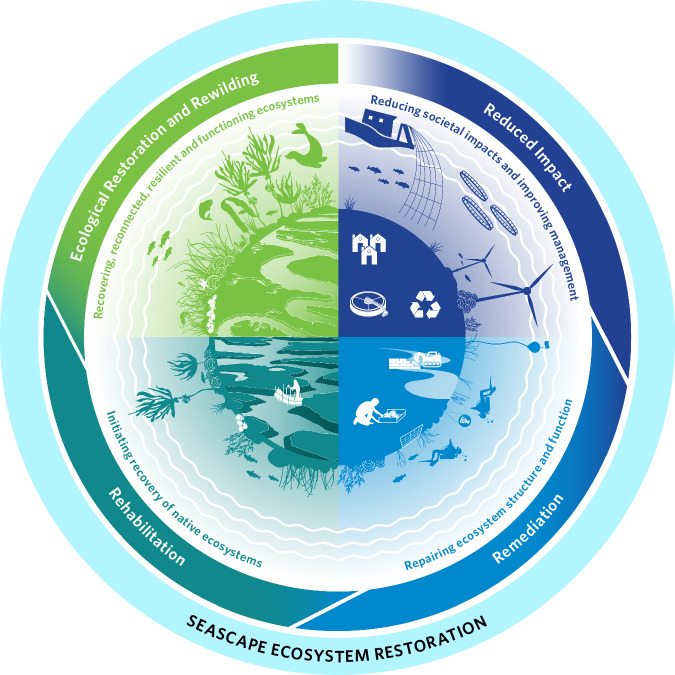
The seascape ecosystem restoration spectrum includes a range of actions that can be taken along the continuum from reduced impact, remediation, rehabilitation, ecological restoration and rewilding to achieve seascape recovery. Adapted from ref. ^315^.

Nature recovery can be achieved in multiple ways, including protection, restoration and, more recently, rewilding. While protection aims to limit impacts and provide a safe space for nature, restoration and rewilding aim to recover ecosystems following degradation from anthropogenic impacts, and despite different approaches, it is argued they can be complementary in achieving societal goals to restore degraded or lost ecosystems^81^ and reverse biodiversity loss^82^. According to their standards and principles, both restoration and rewilding aim for recovery that allows adaptation to environmental change, and that facilitates an ecosystem’s capacity of self-organisation to enable ecosystem resilience^83,84^. Ecological restoration is defined by the International Standards of Ecological Restoration (SER) as ‘*the process of assisting the recovery of an ecosystem that has been degraded, damaged or destroyed*’^83^; and differs from rewilding in defining a target reference ecosystem based on the known or presumed state of a given ecosystem before degradation. There is no globally agreed definition of rewilding^85^, but it can be broadly defined as facilitating self-sustaining, self-organising and resilient ecosystems shaped by natural processes^72^. Rewilding seeks to allow nature to heal itself, restore ecological processes that support trophic webs with apex predators and elicit a paradigm shift in human-nature relationships^84^. Here the term **ecosystem restoration** is used to span this spectrum of restoration theory and approaches, and applied to the seascape context, depicted in Fig. 1.

The connection between people and land/sea is at the heart of successful ecosystem restoration. The relationship humans have with the ‘seascapes’ they perceive will be determined by the character of the seascape, which in turn is influenced by the interactions between biophysical processes and human factors^86^. The term ‘landscape’ has been applied to a variety of contexts, such as landscape planning, or landscape ecology. Here we provide a broader definition of a *coastal seascape* that integrates socio-ecological processes to offer a concept that can be useful for spatial management and recognises the interconnectedness of these marine ecosystems *and* people’s dependence on them for health and wellbeing as part of this complexity and connectivity. The definition and statement below were developed during the symposium and workshop, and subsequently refined through broader consultation with stakeholders. The purpose of the **definition** is to bring to life the seascape concept in a tangible way for non-specialists, and the **restoration statement** aims to advocate an approach to restoration that acknowledges the reality of the ecology of coastal environments and the impact of their dynamic, connected nature on delivery of ecosystem services, restoration goals and ultimately humanity’s wellbeing.

Here we **define the** ***Coastal Seascape*** **as:**

*The mosaic of habitats occupying the coastal environment across time and space* *that are* *ecologically and physically connected via water through which living organisms (e.g., phytoplankton, larvae, fish), genetic material (e.g., seeds, spores, and gametes), non-living matter (e.g., sediment, carbon, nutrients, pollutants) and energy flow*. *This connectivity operates at scales of metres to kilometres and extends from the intertidal zone to the shallow coastal shelf seas. The coastal seascape therefore, acts as a dynamic boundary, where social, marine, terrestrial and atmospheric processes interact, and offers an opportunity to restore and enhance coastal ecosystem integrity and resilience for the benefit of people and planet:*

Seascape composition, scale, condition and spatial arrangement of habitat patches will affect the connectivity and functioning of coastal ecosystems, influencing trophic (food) webs, patterns of biodiversity and ecosystem service flows (e.g., carbon sequestration or denitrification). Acknowledging the interconnected nature of these systems allows for more effective and holistic management, conservation, and restoration strategies:

**Seascape restoration statement**: *Seascape restoration is rooted in the understanding that coastal ecosystems are dynamic and heterogeneous mosaics of habitats interconnected by water through which living organisms, non-living matter and energy flow. The socio-ecological context of a site, habitat configuration and interconnectivity between neighbouring habitat types will shape the outcomes of marine restoration projects. To restore complete trophic webs, enhance biodiversity and ecosystem functioning and deliver ecosystem services requires the existence of a healthy mosaic of coastal habitats, maintained by the flows that occur between them*. *Therefore, a seascape approach that enhances connectivity and restores structure-function relationships is crucial for successful ecosystem restoration*.

**Seascape Restoration:** *restoration of multiple habitats concurrently or sequentially to restore functionality, connectivity and resilience across the mosaic of habitats in a marine ecosystem*.

### Ecological connectivity definitions applied to the coastal seascape

Ecological connectivity, refers to the movement of geophysical, chemical and biological materials across a landscape or seascape^87^ and here we use the definiton from the *Convention on Conservation of Migratory Species (CMS)*^336^:

*The unimpeded movement of species and the flow of natural processes that sustain life on earth.*

Ecological connectivity plays a vital role in maintaining genetic and biological diversity, species persistence, and ecosystem resilience, ultimately influencing the structure and function of marine ecosystems^88–90^. As a concept, it is often split into *structural* and *functional* connectivity components, where the latter is often further categorised as actual and potential functional connectivity^91–93^. Here we use the overall broad categories of structural and functional connectivity, based on the IUCN definitions^94^, adapted to apply to the marine environment:

**Structural connectivity** *describes the permeability of the seascape arising from the physical characteristics, such as spatial proximity or configuration of habitat patches, that confers functional connectivity (e.g., habitat stepping stones that enable organisms to move through the seascape)*

**Functional connectivity** *describes the responses of organisms to this seascape structure and the movements and exchanges that entail. This phenomenon occurs through various types of connectivity, such as larval and seed dispersal, migratory movements of individuals, populations or species, and influences the transfer of non-living matter, such as nutrients, and energy from one location to another*.

**Fig. 2:**
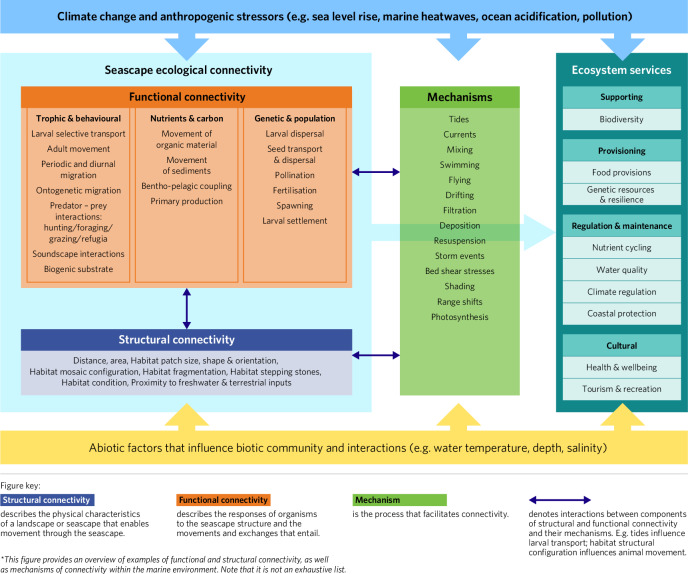
A conceptual framework of seascape ecological connectivity: Examples of structural and functional connectivity are listed, with the mechanisms that facilitate them, and the ecosystem services and functions they underpin.

**Fig. 3:**
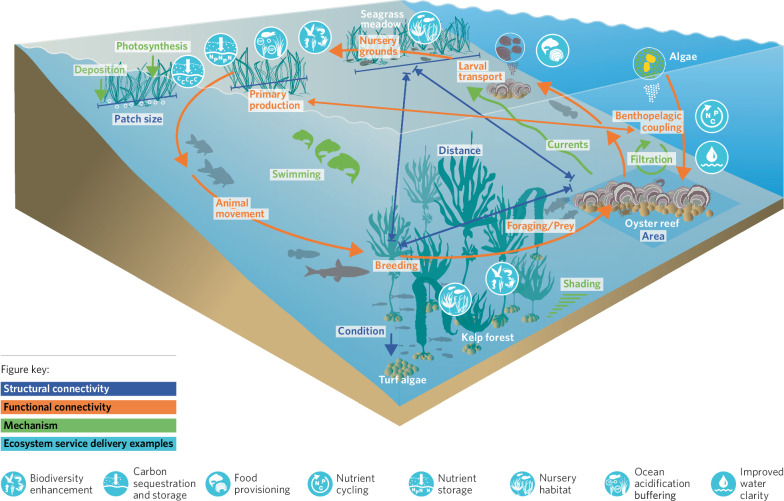
Schematic figure illustrating how structural connectivity, functional connectivity, mechanisms and ecosystem service delivery relate. Examples of structural connectivity are denoted by blue arrows and font, functional connectivity by orange arrows and font and mechanisms are indicated by green arrows and font. The light blue icons provide examples of ecosystem services delivery enhanced by the connectivity across seascape habitats.

The underlying mechanism is the process that facilitates this connectivity, such as water flow, swimming, drifting or active filtration. The conceptual diagram of seascape connectivity in Fig. 2 provides the framework for this paper. It illustrates the relationship between structural and functional connectivity, the mechanisms (processes) that facilitate them, and the ecosystem services and functions underpinned by seascape connectivity. The term *mechanism* is used here as ‘*a natural or established process by which something takes place or is brought about*’^49^ (Oxford English Dictionary). Within the framework, examples of functional connectivity described in the coastal seascapes are grouped into subcategories of animal movement (behavioural and trophic), larval/seed movement (genetic and population) and non-living matter, (nutrients and carbon), and reflect the paper’s structure. The framework is visualised in Fig. 3, which illustrates how structural and functional connectivity interact via mechanisms to deliver ecosystem services and functions. The term ‘Facilitative processes’^52^ is not used here in describing connectivity, as although a helpful concept, these processes can be both mechanisms and ecosystem functions, so are separated for clarity.

As shown in Figs. 2 and 3, a variety of mechanisms mediate connectivity. The scale at which functional connectivity operates will be a product of the spatial scale of the mechanisms that connect related habitats (Table 2).

**Table 2:** Scales of the mechanisms that underpin ecological connectivity across the temperate seascape

**Fig. 4:**
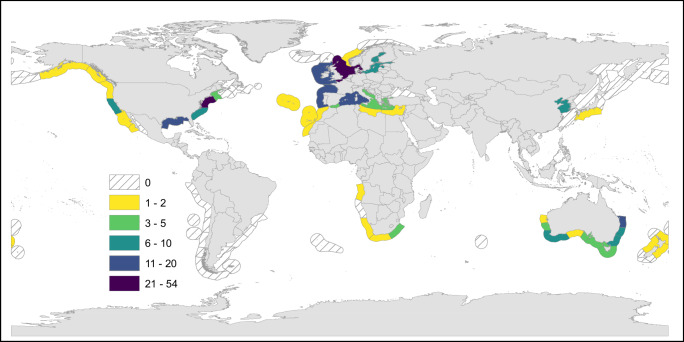
Map illustrating the location of the research study and number of published scientific studies in each temperate ecoregion identified as examining the evidence of connectivity between biogenic coastal habitats (full database is available, see data availability statement). Tropical and polar biogeographic regions are excluded from the analyses. The white and grey hatched area (0 studies) illustrates the substantial evidence gaps in this study across large biogeographic regions, particularly South America and Russia.

Here we provide a ‘state of knowledge’ synopsis of the evidence for ecological connectivity recorded between biogenic temperate coastal habitats (full database is available, see data availability statement), expanding on the framework in Fig. 2. The paper systematically draws out and highlights evidence of ecological connectivity using examples from across the major biogenic habitats. First, we consider structural connectivity (e.g., extent, configuration) and how physical interactions (e.g., hydrodynamics, sediments) affects ecosystem functionality. Second, the functional connectivity responses to the seascape structure are explored through the sub-themes of biodiversity, nutrient cycling and carbon flows. The evidence for ecological connectivity across the temperate seascape is framed within these themes to enable more explicit linkages to the triple planetary crises of biodiversity loss, pollution and climate change, respectively. The distribution and number of research studies contributing to this study shows clear biogeographic evidence gaps and potential biases (Fig. 4), discussed below in the section ‘*Research priorities to address knowledge gaps of temperate seascape ecological functioning and connectivity*’.

### Evidence and impacts of **structural connectivity** on ecological functioning of temperate seascapes

Here we consider how physical processes and structure influence ecological connectivity. Understanding and predicting how oceanographic processes interact with marine ecosystems is crucial for maintaining their functioning. Coastal and nearshore hydrodynamic processes, including currents, temperature, and wave action, play a vital role in connecting habitats and geographic regions by facilitating the movement of organisms, especially those with pelagic life phases. These processes shape connectivity patterns by influencing an organism’s position within the water column, thereby determining the currents they encounter^95–98^. Oceanic fronts, which delineate water masses with differing properties, play a significant role in marine organism dispersal. For example, a previous study^99^ demonstrated how the Celtic Sea Front facilitates the long-distance transport of passive cockle larvae between Britain and Ireland. Conversely, fronts and eddy systems can act as barriers, isolating populations and limiting the exchange of marine organisms between nearby locations. Recirculating eddies and estuarine circulation also influence material dispersal and population connectivity^100^. Estuarine axial convergence fronts can enhance larval retention, which may support the survival of isolated populations through self-recruitment^101,102^. In addition to hydrodynamic processes, the spatial flow of ecological connectivity across landscapes and seascapes is influenced by the composition and spatial configuration of the coastal environment, which is under pressure from anthropogenic stressors.

Physical features such as topographic gradients, habitat patch size, and the presence of barriers (natural or artificial) can all impact connectivity. The interactions of physical dynamics with ecological systems are highly site- and species-specific, emphasising the need for tailored management strategies to preserve and protect coastal habitats. For example, in the UK, saltmarshes, seagrass meadows, intertidal flats, and subtidal habitats are interconnected through annual and inter-annual sediment movement^103–105^. Careful planning and restoration efforts are needed, as sediments can be ‘biostabilised’ by saltmarsh vegetation, microalgal biofilms, seagrass, and subtidal oyster reefs, which dissipate tidal and wave energy^24,106–109^. Conversely, habitat loss in coastal systems increases suspended sediment loading, which in turn reduces primary productivity and disrupts food webs^110,111^.

Some species and processes require continuous habitat corridors, while others can thrive with stepping-stone configurations^42,112^. The geographical proximity of habitat patches can lead to favourable outcomes for species survival and growth by providing access to complementary and supplementary resources, and from cross-ecosystem trophic subsidies^113,114^. In North Carolina, a previous study^115^ found that coastal seascapes with saltmarsh and connected adjacent seagrass exhibited higher faunal diversity and fishery value than seagrass or saltmarsh in isolation. In New Zealand, the presence of kelp forests, and resultant export of organic carbon to neighbouring fjords and inlets, cross-subsidized temperate reef fish density and biomass by supplying basal organic matter^116^. Furthermore, where one patch type meets another, a measurable edge effect often exists with higher diversity and abundance and enhanced predator-prey interactions^64,117^. These varying biological responses to connectivity must be carefully considered when management interventions seek to create or restore contiguous habitats where fragmentation has occurred.

A key benefit of structural connectivity is that it can be physically modified and measured with a wide range of metrics applied to remotely sensed data^118^ making it a pragmatic indicator for restoration projects (e.g., Target 2 of the Global Biodiversity Framework). Spatially representing structural connectivity also enables predictions of biological distributions, material and ecosystems service flows across the landscape and seascape, and the ecological consequences of future scenarios^119,120^. With that in mind, structural connectivity is best understood as a spatial proxy for functional connectivity (Fig. 2) with the caveat that high structural connectivity will be beneficial to some species and processes and less advantageous, or even detrimental, to others^121,122^.

In addition to the substantial habitat loss described above, a key challenge in managing today’s temperate seascapes is the widespread proliferation of hard artificial structures (‘ocean sprawl’). Contributing to habitat fragmentation, this ocean sprawl is altering marine ecosystems by inducing genetic changes, shifting species distributions, and facilitating the spread of invasive species. For example, in tidal marshes and seagrass ecosystems, disturbances—including human activities—can fragment the spatial arrangement of biophysical structure, disrupting or reducing structural connectivity^61^. This ocean sprawl, compounded by altered coastal dynamics under climate change, undermines natural connectivity and ecological functions, reducing population resilience and affecting species’ range expansions^123^. When species expand into new environments, they can destabilise native community structures and ecological functions, with detrimental consequences^124,125^.

Assessing the relative significance of different hydrodynamic and physical forces in shaping marine ecosystems and habitats^103,104,126,127^ is crucial for interpreting ecosystem responses and enhancing resilience. Understanding dispersal within coastal and estuarine regions is an ongoing challenge^95,128–130^.These areas are characterised by complex flow regimes driven by rivers, waves, tides, and geomorphology, meaning representative in-situ measurements of organism dispersal is often not possible. Biophysical modelling has become an invaluable tool for studying population connectivity, particularly when combined with empirical seascape genomics data^99,131–134^. However, observational data on biological behaviour, such as larval swimming and diel vertical migration, remain limited, yet are essential for accurately parameterising dispersal models and predicting connectivity patterns. Future research should work to integrate observational data with bio-physical modelling.

Despite advances, structural connectivity has tended to focus on simplistic and static representations often failing to capture the dynamics and directionality of responses. Structural connectivity and functional connectivity interact to shape each other in complex ways making it important yet challenging for studies to consider feedback between process and structure (e.g., hydrological networks in saltmarsh dynamics^135,136^). Indeed, some structural connectivity metrics have been modified to include species-specific information (e.g., habitat suitability) or ecological processes (e.g., dispersal distances), bringing them closer to functional metrics^91,118^. These advancements in structural connectivity metrics enhance their utility for conservation planning and management by better accounting for species-specific needs and ecological processes. Further work is needed to develop structural connectivity metrics that provide appropriate proxies for a wider suite of functional connectivity across temperate habitats.

The importance of connectivity (inflows and outflows) across the land-sea continuum means that in some cases restoring or enhancing coastal connectivity may require whole system consideration of both horizontal and vertical linkages from land (e.g., summit to sea, ridge-to-reef) to intertidal and deeper subtidal habitat^137,138^. An ecosystem-based approach is essential for managing anthropogenic stressors on temperate seascapes, as alterations in marine structural connectivity can have far-reaching ecological consequences.

### Evidence of functional connectivity across habitats in the temperate seascape with implications for trophic webs and biodiversity recovery

The rapid degradation and loss of biodiversity in coastal and estuarine ecosystems over the last 150-300 years has had profound impacts on trophic connectivity and ecosystem functions^15^. In particular, widespread losses of habitat-forming species such as saltmarsh, seagrass, kelp and biogenic reefs have fragmented the temperate seascape, while massive declines in apex predators - largely due to overexploitation - have impacted food web structure and key ecosystem services^27,35,139–142^.

Vegetated habitats such as saltmarsh, seagrass and kelp forests provide significant benefits in the form of coastal protection^143^, bioremediation^144^, CO_2_ drawdown^145^, biodiversity maintenance^146^, and environmental buffering^147^. These complex habitats also provide critical nursery areas, defined as providing increased productivity per unit area, thereby supporting many coastal fisheries^148–150^. For example, 45% of kelp primary production is estimated to remain in UK coastal waters^151^, supporting local high biodiversity^146,152^. The benefits of enhanced fisheries resilience are magnified with increased connectivity between saltmarsh-seagrass-kelp^153^ enhancing ecosystem productivity, fishery biomass and energy flow^154^, and increasing trophic complexity and ecosystem resilience^155,156^. However, climate change, coastal development, nutrient runoff, and destructive activities have led to significant degradation and loss of saltmarsh, seagrass, and kelp forests^23,26,157^, with losses from UK coastlines estimated at 90%, 44% and >30% within the last century, respectively^104,141,142^. This large-scale habitat loss has significantly affected ecosystem structure, function, trophic complexity and connectivity^158,159^. For example, the loss of kelp forests (and corresponding loss of habitat complexity) has been linked with a 90% loss of associated macrobenthic communities^35^. The flow of organic carbon from macroalgal habitats to neighbouring coastal water bodies supports fish population biomass and density via the contribution of basal organic matter, which can have negative impacts on communities when removed due to habitat loss^116^.

Shellfish reef habitats provide many empirical examples of the importance of trophic connectivity in underpinning biodiversity and ecosystem resilience (see also Supplementary References 2). Numerous fish species use shellfish reefs as juveniles as a food source or as refuge, even in areas where the habitats are found intertidally^160^. These reefs remain important foraging grounds for many transient species during later life history stages, with species such as Black Drum (*Pogonias cromis*) preying directly on oysters within reefs on the Atlantic coast of the US^161^. In some species, this specialised feeding appears mediated by the functional connectivity and habitat types present within the seascape. For example, while cownose rays (*Rhinoptera bonasus*) are widely cited as being important predators on bivalve molluscs in many estuarine settings^162^, their prey preference is known to differ greatly depending both on the locale and with prey availability^163^. Importantly, it is not only through predator-prey interactions that shellfish reefs are connected to the seascape, with the soundscape associated with healthy *Ostrea angasi* reefs in South Australia also having been shown to play a key role in attracting oyster larvae from the water column^55^. The growth of oysters within the reef system acts to support further trophic connections through the provision of substrate for a wide range of epibenthic species^164^. The importance of shellfish reefs for biodiversity is particularly stark given that flat oyster *Ostrea edulis* reefs, once a dominant three-dimensional feature of European coastlines occupying over 1.7 million hectares and supporting a rich biodiversity^18^, no longer exist at ecosystem scales in Europe^30^.

Loss of keystone species can cause top-down changes to the ecosystem structure and functioning of the coastal seascape, with overfishing of apex and mesopredators associated with large-scale trophic cascades^165,166^. For example, there are many examples of shark overfishing being linked to predator release of herbivorous species, leading to loss or slower recovery times of seagrass habitats^140,167,168^. In temperate systems, the overfishing of species such as cod and herring has resulted in trophic cascades that have directly impacted the coastal environment by disrupting their movements inshore for feeding or spawning, or indirectly impacted the environment through linked trophic connectivity pathways. For example, the loss of cod from the Gulf of Riga in the Baltic Sea resulted in predator release for herring, with the subsequent increases in herring associated with decreases in their zooplankton prey and basin-wide increases in phytoplankton^169^. Overexploitation of cod and otters has also been linked to rapid proliferation of inshore herbivorous species such as urchins, widespread decimation of kelp forests, and subsequent losses of kelp-reliant species such as the Steller’s sea cow, *Hydrodamalis gigas*^170,171^. Overfishing can also lead to declines in another fishery, with the collapse of the Norwegian spring-spawning herring *Clupea harengus* in the 1960s and associated loss of coastal egg boons thought to have caused large declines in the lobster *Homarus gammarus*^172^. Trophic cascades can also be triggered by overfishing within coastal environments, for example with intensive recreational fishing of predators such as striped bass (*Morone saxatilis*) and smooth dogfish (*Mustelus canis*) associated with increased abundance and grazing rates of the herbivorous *Sesarma* crab, and widespread loss of saltmarsh vegetation^173^.

### The role of mobile fauna in facilitating ecological connectivity across the seascape

Ecological connectivity is central to the functioning of coastal seascapes, supporting mobile fauna species by facilitating individuals to meet their basic needs on daily, seasonal, and lifecycle timeframes^68^. Impacts to connectivity, such as the degradation of habitat quality or condition^174^ and barriers to migration^175^, can undermine the sustainability of populations that use coastal seascapes^176^. Fish movements through the coastal seascape mediate connectivity across a broad range of spatial and temporal scales, from daily tidal foraging migrations^177^, through seasonal and ontogenetic habitat shifts^70^, to life-cycle migrations from coastal nurseries to adult feeding and spawning sites that may be 10’s, 100’s, or 1000’s of km away^178^.

At fine spatio-temporal scales, many species move among multiple habitats within the seascape daily to access prey resources^177^, avoid predation, or seek out favourable environmental conditions^68^. Foraging movements among habitats by individuals who are subsequently eaten by predators forms a trophic relay that transfers energy and production across the seascape facilitating the trophic support of predators in one habitat by production from another^69^. The arrangement of habitats within the seascape can also have significant effects on individual fitness, such as enhanced fish growth when productive intertidal and subtidal habitats occur immediately adjacent to each other^179^. The edges of individual habitat patches often contain higher densities of aquatic organisms^117^, so the size and arrangement of individual habitat patches within the seascape can strongly regulate the movements of fish^180^.

At moderate spatio-temporal scales, many species undergo predictable ontogenetic shifts among habitats within the coastal seascape^70^. The value of habitat patches depends on their position within the seascape, such as variation in community composition among seagrass meadows depending on their position within the estuary relative to the estuary mouth and tidal channels that deliver larvae^181^. The distance between habitat patches is also important. In tropical systems, the distance between coral reefs, seagrass meadows and mangrove forests impact the fish communities found in those habitats^182–184^. Additionally, contextual factors such as tidal range, rainfall and seascape composition can have strong influences on the use of particular habitats^185^ and can regulate energy flows across the seascape^186^.

The role of coastal seascapes as nursery grounds facilitates the flow of energy across large spatial scales. While the spawning migrations of salmonids provide some of the most famous examples of large-scale transboundary nutrient flows^187,188^, the life cycles of all species that move between coastal seascapes and other ecosystems are examples of large-scale connectivity. Many fish species form large spawning aggregations off the coast^189^, and the subsequent inshore drift of millions of eggs and actively feeding larvae represents a significant nutritional resource for estuarine and coastal species. These ‘egg boons’ can create large, spatially discrete pulses of food to consumers^190^, with a single population of Norwegian herring depositing about 1.3×10^6^ tonnes of biomass from their reproductive outputs, thought to be the world’s largest flux of energy caused by a single population^172^. Fish-mediated resource flows also occur in an offshore direction, with juveniles and sub-adults of many species spending years accumulating body mass in estuarine and coastal nursery areas before moving offshore as they mature^32^. These fish-mediated connectivities across coastal seascapes at multiple spatial and temporal scales highlight both the importance of fish movements in facilitating the flow of energy through the seascape, as well as the critical need to maintain this connectivity to sustain fish populations.

### Evidence of functional connectivity across habitats in the temperate seascape with implications for nutrient cycling and pollution mitigation

Nitrogen pollution poses a serious threat to coastal and estuarine ecosystems worldwide^191–193^. Particularly in industrialised, temperate nations, combined pressures of sewage discharge, fossil fuel burning and land based agricultural run-off has resulted in a dramatic increase in nutrients, and particularly nitrogen, entering coastal systems^194–196^ leading to eutrophication (excessive nutrients in coastal waters), a phenomenon compounded by the loss of wetland habitat^197^. Nutrient concentrations decline with distance from source, due to abiotic processes (i.e., flocculation), biological utilisation, and dilution with seawater (conservative mixing). This attenuation results in gradients of nutrient concentrations across a coastal seascape, dependent on salinity and water flows^198^.

Nutrient bioremediation is a key ecosystem function that improves water quality and prevents nuisance and harmful macroalgal and phytoplankton blooms, which impact fisheries and human health^24,198–200^. Coastal habitats can play an important role in bioremediating this pollution. When nutrients are captured in biomass or sediment for long periods of time, it is considered burial or storage^201^. Biological denitrification permanently removes the biologically available form of nitrogen that causes algal blooms^202^. The degree of nutrient bioremediation is strongly influenced by the structural connectivity of habitats within the seascape, due to their differing influences on hydrology and therefore abiotic processes, and their variable influence on coastal sediment processes. For example, coastal sediment nitrogen biogeochemical cycling is complex, influenced by biological actors across scales from the microbial to megafauna, and driven by nitrogen availability and concentration, temperature, oxygen concentration, water depth, organic matter quality and quantity, bioturbation and turbidity levels^203,204^ which vary and interact in diverse ways across a seascape. Below, we summarise some key examples of connectivity relating to nutrient cycling in temperate coastal seascapes by considering the biogenic and connecting habitats.

Unvegetated sediments interconnect coastal seascapes and play an important role in nutrient cycling, primary productivity, sediment supply and wave and tidal flow attenuation^204,205^. For example, mudflats connect intertidal and subtidal habitats and provide a source of sediment to adjacent vegetated saltmarshes^206,207^. Occupying large areas, mudflats, mixed flats and subtidal sediments have significant impact on nutrient attenuation^201,208,209^, and the greater the area of intertidal and subtidal sediment, the more nutrient attenuation can occur^210^. Mudflats also exhibit high rates of biogeochemical cycling and primary production, significantly influencing both carbon and nitrogen fluxes in shallow-water systems^208,211–213^. For example, over 35% of the nitrogen load in the Colne estuary UK, is removed by sediments^204,208,214^. Whilst eutrophication may enhance nitrogen removal to mudflats through increased burial of green macroalgae^201^, across the wider seascape smothering by green macroalgae can compromise nutrient removal (and other ecosystem functions) of other habitats^215^. In contrast, impacts on nutrient attenuation rates by other anthropogenic activities (e.g., physical disturbance, such as dredging) remain understudied^216^, though attenuation of wave and tidal energy by adjacent seagrass will reduce physical disturbance^217^.

Limited data on nitrogen cycling in saltmarsh sediments, and connectivity to other habitats exists for many otherwise well-studied regions, such as the UK. Although the underlying microbiological drivers remain, limited time periods of tidal cover (providing external N supply), and the presence of aerobic sediments can reduce the potential for denitrification^208^. Detailed biogeochemical studies of saltmarshes (Colne Estuary, UK) found an overall neutral annual N budget, with export of ammonium, dissolved organic nitrogen (DON) and large size particulate organic nitrogen (PON) (particularly in summer) balancing import of total oxidised nitrogen (TOxN), and small size PON^208^. However, reliable extrapolations to overall saltmarsh annual nitrogen budgets are limited especially as decomposition of detrital wrack of seagrass, macroalgae, and terrestrial inputs can result in pulses of nutrients released to marshes^218^. Nevertheless, temperate saltmarsh denitrification (25.2 g N m^−2^y^−1^) and burial rates (10.8 g N m^−2^y^−1^), was found to be higher than in seagrasses (15.1 g N m^−2^y^−1^ and 4.9 g N m^−2^y^−1^) for Northern Europe^201^, with higher denitrification rates reported in US coastal marshes^219^. Saltmarshes can therefore provide a protective service to nutrient-sensitive seagrass meadows^219^. Comparisons must be made with care, due to geomorphological and floral differences between European and North American saltmarshes. Periods of tidal cover (bringing in allochthonous nitrate) and extent of saltmarsh (due to historic habitat loss) are much lower in the UK^201^, meaning internal (re)cycling of N from organic matter processing may be more significant.

As filter feeders, bivalves, such as oysters, consume phytoplankton and organic suspended matter from the water column^202^, providing the potential for bioremediation of eutrophication in coastal waters^220–222^). Through nutrient assimilation, bivalves convert the food they consume into biomass (tissues/shells), and if they form biogenic benthic structures and in sufficient densities, can trap and stabilise sediment^206^. In addition, both through bentho-pelagic coupling via the mechanism of waste excretion/deposition, and by providing a substrate for microbial colonisation, biogenic shellfish habitats can enhance denitrification^223^. Filter-feeding bivalves can therefore significantly influence the coastal nitrogen cycle and offer a potentially powerful tool for improving water quality and clarity in coastal and estuarine ecosystems. However, the habitat to which they are connected provides an important context.

The interactions between shellfish reef characteristics (e.g., oyster biomass, associated macrofaunal assemblage, infaunal communities; structural complexity), and the environmental factors within the seascape (e.g., tidal regime, light regime, water depth, temperature, salinity, availability of nitrate, quality and quantity of available organic matter) have a significant influence on nitrogen removal by oyster habitat^75,197,224,225^ making it an extremely complex process to investigate^226^. Given the collapsed status of native oyster reef ecosystems in European waters^30^, it is unsurprising there are no in situ studies to assess nutrient flux in *Ostrea edulis* habitat, and few mesocosm laboratory studies exist on the nutrient cycling by the *O. edulis*^226^. Work on the temperate reef building *Crassostrea virginica* in the US found enhancement of denitrification by 18–275% on restored reefs compared to unvegetated mudflats, although denitrification was 4% lower in oyster reefs co-located with saltmarsh or seagrass compared to reef-mudflat associations^224^. In North Carolina, a weak positive effect of oyster presence on sediment denitrification in associated habitats (seagrass, saltmarsh and mudflat) has been reported^224^. Site characteristics were an important driver of the coupling nitrification-denitrification processes of *O. angasi* shellfish reefs in Australia, with denitrification and nitrogen flux strongly influenced by sediment types, with greater denitrification observed in oysters on sandy sediment, but net nitrogen efflux in areas with fine sediments^41^.

In the context of connectivity, healthy seagrass meadows likely positively influence the wider seascape by removing nutrients from the water column. Seagrass mediated nitrogen cycling is the result of high productivity and consequent plant uptake of nutrients but is also due to an active and unique microbiome assemblage associated to their root tissues that contains nitrogen cycling bacteria performing nitrogen fixation, nitrification and denitrification roles^227^. The fungal community is also quite prolific in these roots and likely to play a major role in such cycling but little is known of its role or identity^228^. Seagrass restoration in the US resulted in nitrogen removal via burial twenty times higher than removal to adjacent mudflats^229^), and recent methods report nitrogen fixation rates of 250 - 550 umol N_2_ m^−2^ h^−1^^230^. Seagrass (*Zostera*) meadows associated with oyster reefs in Baja California uptake excreted ammonium, resulting in increased shoot size and leaf growth compared to un-associated *Zostera*, which allocated more below-ground growth to facilitate the uptake of sedimentary nutrients^231,232^. A study^233^ found that oysters increasing sediment carbon and porewater ammonium did not affect associated *Zostera*, but space competition did. Patch density affects root density, affecting oxygen concentrations in the sediment and thus the microbial communities^234,235^. During the decline period of *Zostera noltii*, large exports of dissolved inorganic phosphorus to the water column and porewater of neighbouring unvegetated sediment in Archachon Bay, France^236^ and the Mondego estuary, Portugal^237^ have been recorded. Detrital seagrass is refractory, which reduces consumption rates and allows accumulation as macroalgae wrack in other habitats (e.g., saltmarsh or mudflats)^238^.

Nutrients do not move unimpeded through the coastal marine environment, but in nutrient spirals analogous to river systems^239^. The actions of biological habitats and species slow down and facilitate connectivity. Nutrient sinks are areas where the speed of movement of nutrients is slowed. Understanding context dependency and habitat configuration within seascapes to enhance nutrient cycling provision is key. Modelling for particular seascapes is required to determine the role of seascape connectivity in the overall potential for nutrient attenuation, where limited naturally-functioning ecosystems exist (e.g., European oyster *O. edulis)*.

### Evidence of functional connectivity across habitats in the temperate seascape with implications for carbon storage and climate resilience

The ocean is our planet’s major carbon sink^240^, and therefore provides a critical climate regulating function, having absorbed over 25% of all total anthropogenic CO_2_ emissions to date^241^. In addition to this climate buffering, marine sediments represent a long-term carbon sink and store. Coastal sediments are at the front line of this organic carbon sequestration and storage (CSS); given blue carbon habitats represent 0.2% of the seabed, yet contribute 50% of the total carbon sequestered and stored in ocean sediments^242–244^. While the term “blue carbon” was originally applied to CSS in vegetated coastal habitats (mangroves, saltmarshes and seagrass meadows)^243,245^, it is now used to refer more broadly to coastal and oceanic carbon and has been expanded to include other habitats (e.g., macroalgae, oyster reefs, tidal flats and maerl) and the role of pelagic fauna (e.g., whales)^246–249^.

The movement of organic carbon across the seascape may be multidirectional, with habitats acting as both donors and receivers (Table 3). Physical processes such as tidal exchange and vertical settling can lead to this multidirectional movement, such that the resuspension of particulate organic matter (POC) in the water column creates movement of carbon between habitats (e.g., microphytobenthos, saltmarsh and seagrass)^250^. These directional transfers of carbon are not uniform, but exhibit daily and seasonal patterns, such as higher suspensions of microphytobenthos at the beginning of the flood tide within a typical tidal cycle and yearly maximums during spring tides^251^. Likewise, receiver habitats may not represent significant carbon storage sites; upper intertidal sediments receive large imports of carbon in the form of wrack or detritus from coastal habitats, most of which is likely remineralised (Table 3, references therein).

**Table 3:** Conceptualisation of organic carbon donor, receiver and storage (shaded grey) habitats/components across the temperate seascape

Seascape habitat / component	Donor habitats / components	Receiver habitats / components
Estuarine & marine phytoplankton / Suspended Particulate Organic Matter (SPOM)	Contributes to organic carbon within the seascape, but contribution to a habitat decreases with elevation: offshore > macroalgae > seagrass > saltmarsh^252,253^	
Saltmarsh	Can act as a source to adjacent habitats, connected by hydrodynamic lateral and vertical transport mechanisms (e.g., resuspension, tides/currents, sinking)^250^	Upper marsh can show slower sedimentation due to lower tidal inputs. Lower marsh often demonstrates rapid accretion of allochthonous inputs e.g., Particulate Organic Matter (POM)^250,256^
Microphytobenthos (MPB)	MPB originating carbon found in deposit feeding bivalves, so MPB carbon can move through seascape via vectors. Also resuspended seasonally leading to further movement through the seascape^212,251,254,255^	
Seagrass meadows	Export of organic carbon to sublittoral reef habitats, and sublittoral or intertidal sediment, linked to hydrodynamic transport. Also deposited in the upper intertidal as wrack^127,256–260^	Microalgae and POM donated to seagrass. Saltmarsh vegetation exported offshore to subtidal seagrass^250,256^
Macroalgae seaweed bed (excluding kelp)	Export of carbon to intertidal and offshore sediments, linked to hydrodynamic transport mechanisms^257–262^	Evidence of POM within macroalgae habitats, particularly for species (e.g., Caulerpa spp.)– which demonstrate rhizoidal growth^252^
Intertidal / sublittoral / offshore sediments		Receives carbon via SPOM / phytoplankton, seagrass (as wrack) and macroalgae (as wrack). Evidence that sediment below organism's burrows in sediment are enriched with macroalgae debris^253,256–261,263^
Sublittoral reefs		Receives organic carbon from seagrass and macroalgae^256^

The direction, and where possible the mechanisms, of carbon movement are given. The evidence was extracted from the literature scoping review, focusing on those sources categorised as providing evidence of carbon flows within the temperate seascape. As such, we recognise the collated evidence is not comprehensive, given the large body of literature examining organic carbon within individual habitats / components. Where the table is left blank no evidence to demonstrate carbon donor, receiver or storage within that seascape habitat or component.

In addition to organic carbon from vegetated habitats, seascape fauna contributes to and influence CSS, both directly through the standing stock of organic carbon in tissues, and indirectly through a wide range of functional roles. While studies estimating the faunal contribution to organic carbon storage in the seascape are currently limited, in the polar seascape of the Antarctic Peninsula, zoobenthos stocks, productivity, and contribution to sequestration were found to be significant in comparison to that from vegetation^252^. For example, benthic invertebrates’ organic carbon standing stock was around twice that of macroalgae in rocky areas, and around three times greater in areas with mixed substrates^252^. Furthermore, total standing stock and sequestration rates were observed to increase as functional diversity increased^252^.

As previously discussed above, one of the most dramatic examples of functional control is the removal of keystone predator species leading to trophic cascades and the degradation of vegetated ecosystems. Such that the presence of sea otters at ecologically effective densities enhances ecosystem carbon production and storage by a factor of around 12–13^253^. Similarly, in the North Atlantic, removal of predatory fin fish and crabs (e.g., striped bass, *Morone saxatilis*; smooth dogfish, *Mustelus canis;* blue crab, *Callinectes sapidus)* through recreational fishing resulted in a population explosion in herbivorous saltmarsh crabs *Sesarma reticulatum*^173^. This not only increased grazing pressure, but also increased burrowing, leading to extensive loss of saltmarsh through bank calving, and thus the loss of both a carbon sink and store^197^.

Depending on seascape context, shellfish beds and reefs may represent net sources or sinks of atmospheric carbon dioxide, reflecting the balance between inorganic carbonate precipitation (the net effect of which is to release carbon dioxide) and organic CSS^254^. For example, experimental oyster reefs situated on intertidal sandflats were dominated by carbonate precipitation and acted as net sources, whilst shallow subtidal and saltmarsh-fringing reefs were dominated by organic rich sediments and sequestered carbon at a rate comparable to vegetated habitats^254^.

Reef-builders can enhance carbon sequestration through multiple mechanisms, including synergistic effects on adjacent vegetated habitats. Shellfish suspension feeding can enhance light availability and thus seagrass productivity^255^, while production of pseudofaeces and faeces can enhance transfer of organic matter to the sediments^256^. For this reason, shellfish reefs can be considered enabling habitats in relation to carbon sequestration and storage by submerged aquatic vegetated habitats in light limited environments. Though there is limited experimental evidence, history indicates that seagrass decline approximately mirrored the overexploitation of oysters in many locations. Historic herbarium specimens of seagrass in the Firth of Forth in Scotland from the 1800’s have shoot lengths of up to a metre^257^, a time when oyster reefs of the Forth were extensive, but by 1885 oysters were almost non-existent^258^. Since then, seagrasses have declined, and remaining plants are short-leaved populations living in the margins^257^. Furthermore, bioconstructions such as reefs can influence hydrodynamic and geomorphological processes through bioprotection^259^, creating depositional environments where soft sediments can accumulate and become colonised, thereby further enhancing organic carbon sequestration and preservation (e.g., seaward expansion of saltmarsh in the Rachel Carson Reserve^254^.

While shell and skeletal carbon is not a sink of atmospheric carbon dioxide, interactions between the inorganic and organic carbon systems may confer carbon benefits. The production of calcium carbonate can make a significant contribution to sediment accretion rates (accelerating organic carbon burial), the presence of calcium carbonate in sediments can both inhibit microbial activity and carbonate dissolution could mop up carbon dioxide released during remineralisation of organic matter, transferring POC to the dissolved inorganic carbon (DIC) pool^260^ The presence of healthy vegetated ecosystems may also confer benefits to in-fauna and adjacent communities through localised amelioration of ocean acidification, where photosynthesis driven lowering of pCO_2_ in seagrass meadows is to the benefit of proximal shell (and skeleton) builders^261^. In some case studies this has been found to occur at the scale of the whole coastline^262^

### Interactions across habitats and stressors with implications for CSS and carbon accreditation frameworks

In addition to the examples noted above, biogeomorphological processes can interact with other stressors with implications for climate resilience and CSS within the seascape. In tightly coupled and highly dynamic saltmarsh-mudflat-seagrass systems, vegetation communities reflect elevation, and the ability to build elevation (through sediment trapping and organic matter accumulation) to keep pace with sea-level rise depends on vegetation, where feedback mechanisms between different habitats can influence the evolution of the entire system^263,264^ For example, modelling suggests that the presence of seagrass can either increase or decrease the resilience of saltmarsh-mudflat systems to sea-level rise, depending on the geometry of the system^263^. There is evidence that some NbS such as bivalve or artificial reefs, saltmarsh or coastal wetland restoration^108,110,265,266^ may alleviate some of the impacts of coastal flooding and/or erosion.

Eutrophication has consequences for carbon cycling, influencing the ability of saltmarsh to keep pace with sea-level rise (and thus has implications for the preservation of existing carbon stores). Excess nutrients have differing effects on above and below ground biomass, where the relative importance of differing mechanisms has not been fully elucidated and the net effect may depend on elevation^267,268^. Aboveground, increased stem density can boost sediment trapping, leading to enhanced accretion, while belowground the development of weaker and less extensive root systems can leave the intertidal marsh vulnerable to erosion. Smothering by wrack (e.g., *Ulva* blooms) can also disturb saltmarsh plant growth, an effect often limited to the low marsh. In addition, nitrate pollution can increase litter decomposition by stimulating denitrification, altering the microbial community towards groups able to oxidise more complex organic matter that would otherwise be stored within the sediment^269^.

In existing carbon credit standard methodologies based on single habitats (e.g., VM0033^270,271^), it is necessary to deduct the allochthonous mineral associated carbon to determine the net increase in autochthonous organic carbon which originates from climate mitigation activities such as habitat restoration. Adopting a broader seascape boundary which includes multiple habitats and the flows of organic carbon between them enables multiple organic carbon sources to be viewed as autochthonous in origin^272,273^.To enable this approach, it is necessary to understand the connectivity and flow of carbon across the seascape to identify donor and receiver components or habitats (Table 3) and how structural and functional connectivity influences CSS services.

### The role of connectivity in modulating ecosystem service delivery

**Fig. 5:**
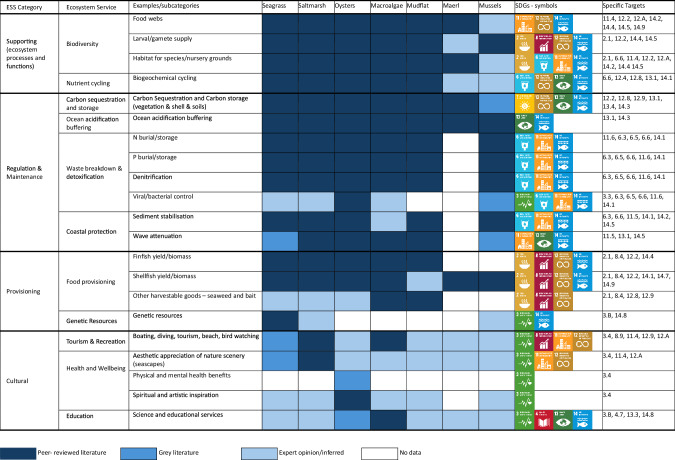
Evidence for Ecosystem services delivery across priority temperate coastal biogenic and connecting habitats, linked to the Sustainable Development Goals and specific targets they support. Supporting references are provided in Supplementary References 2. Coloured boxes denote the source of evidence. Dark Blue: peer-reviewed literature. Medium blue: grey literature. Light blue: expert opinion/inferred. Blank: no data.

Coastal marine benthic habitats provide a wide suite of valuable ecosystem services^274,275^. The evidence for service delivery by each of the major temperate biogenic and connecting habitats collated from the systemic review (Fig. 5, Supplementary Reference 2), demonstrates the substantial value they provide to human wellbeing, as highlighted by the multiple SDG goals and targets they support. It is clear that a diversity of habitats across a seascape therefore provides a corresponding diversity of ecosystem service values, relative to single habitats alone^201^, especially where the ecosystem services provided are complimentary, or where the habitats have different ecological niches.

**Fig. 6:**
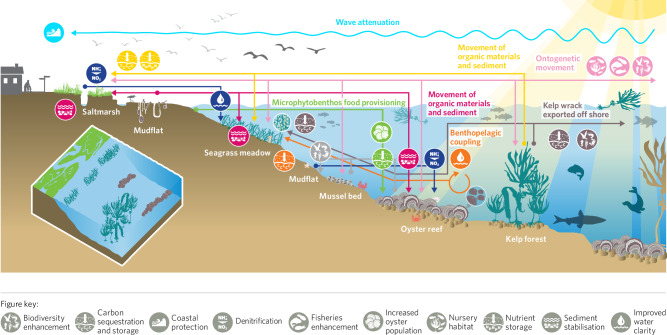
Illustration of the role of connectivity in modulating ecosystem service delivery across the coastal seascape. Arrows relate to icons of the same colour, with the arrowhead indicating the habitat in which the ecosystem service is enhanced through connectivity with the source habitat. LIGHT BLUE: Wave attenuation by habitats increases coastal protection and increases sediment stabilisation^280,341^; YELLOW: Movement of organic materials and sediment from between habitats increases N and C storage. Saltmarshes near seagrasses sequester more carbon and nutrients relative to saltmarshes away from seagrass meadows, by trapping seagrass detritus^278^ ; LIGHT PINK: Animal movement between habitats enhances biodiversity, nursery function and fisheries production^160,342^; DARK PINK: The presence of habitats increases sediment stabilisation^265,280,282^; GREEN: Benthic chlorophyll is a food source for oysters. Where benthic chlorophyll levels are higher, oysters have greater biomass and their filtration draws down more carbon^343^; BROWN: macro algae drifting into seagrass meadows increases the biodiversity associated with the seagrass^279^ and in adjacent deep sea areas^344^; GREY: Infaunal abundance increases with distance from oyster reefs, while the density of large predatory crustaceans is greatest on mudflats near oyster reefs^280^; DARK BLUE: Denitrification mediated by saltmarsh increased water clarity and primary production and C sequestration in seagrass^219^; ORANGE: Benthopelagic coupling by mussels increases the availability of nutrients for seagrass growth thereby increasing seagrass growth^345^, filtration by oysters also reduces incidence of seagrass wasting disease^261^ (full database is available, see data availability statement).

Ecosystem service provision arising from individual habitats have been subject to varying degrees of research (Fig. 5), with habitats often viewed in isolation. Connectivity between habitats can, however, also impact the degree of ecosystem service provision, for example, by providing alternative habitats for different life history stages^276,277^. The vast majority of recorded accounts of connectivity between habitats affecting the provision of ecosystem services result in higher levels of ecosystem service delivery, as illustrated in Fig. 6. For example, seagrass meadows near saltmarshes potentially sequester more carbon and nutrients relative to saltmarshes away from seagrass meadows, by trapping seagrass detritus^278^, and macro algae drifting into seagrass meadows increases the biodiversity associated with the seagrass^279^.

**Fig. 7:**
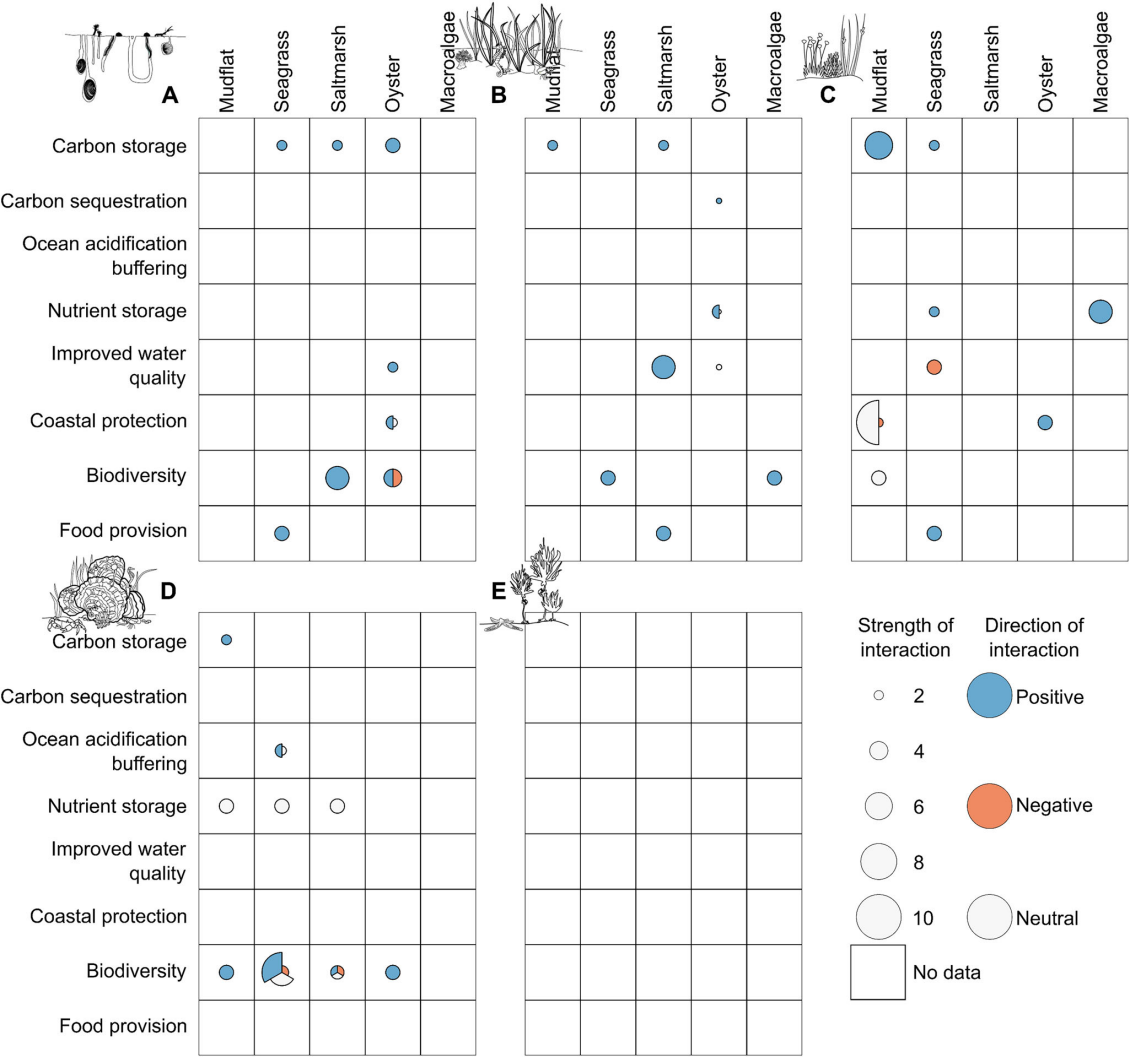
Matrix of ecological connectivity across coastal temperate habitats and its effect on ecosystem service delivery. The strength of evidence for connectivity between each pair of coastal habitat types as having a positive, negative or neutral impact on ecosystem service delivery. Empty boxes represent a lack of data, so interactions are unknown. Where evidence exists, the colour of each segment indicates the direction of the interaction, while the size represents the strength of the evidence, as determined by the sum of studies, weighted by the nature of the evidence with inferred = 1, indirect evidence of a process occurring = 2, quantitative = 3). There is a matrix for each habitat type in which the change in ecosystem service delivery was measured, with matrix A representing Mudflat, B, Seagrass; C, Saltmarsh; D, Oyster; and E, Macroalgae.

Habitats within the seascape can, however, interact in complex ways to modulate the flow of ecosystem services, with different components of the food web responding differently to connectivity, and therefore correspondingly mixed impacts on their related ecosystem services (Fig. 7). For example, oyster reefs have been shown to result in increased abundance of crabs, but these predators also correspondingly cause declines in infaunal bivalves surrounding the reefs^280^, while the degree of predation by crabs may be mediated by connectivity with seagrass and saltmarsh habitats^281^. As both clams and crabs are the subject of commercial fisheries, whether the connectivity between oyster reefs and their surrounding mudflats are viewed as an ecosystem service gain is the social context, such as which species has greater cultural value or economic value to the local coastal community. What is clear, is that the role of connectivity in modulating the delivery of ecosystem services from a habitat can be highly context and scale-dependent. Even starker, are the limited data on the relationship between ecological connectivity and ecosystem service provision in temperate seascapes (Fig. 7).

The impact of connectivity is context dependent, for example, how effective oyster reefs were at preventing erosion on mud flats was related to whether the mud flats themselves were eroding or accreting^282^, while the value of habitats as fish habitat is also dependent on their setting across the land-sea and estuary-open coast interface^283,284^. This variability across abiotic clines in the seascape also affects the community associated with single habitat types, as, for example, seagrasses with different habitat qualities support communities distinct from one another^279^. Even in settings where habitats do not appear to provide added ecosystem service value as a result of their ecological connectivity^285^, they may still provide benefits. Connected habitats can contribute redundancy and increase resilience under pressures such as climate change^286^, due to the multiple ecosystem services delivered by a healthy and heterogeneous seascape (Fig. 5).

### Research priorities to address knowledge gaps of temperate seascape ecological functioning and connectivity

The importance of ecological connectivity has long been recognised by ecologists^93,287,288^ and fundamentally underpins many key ecological theories and processes such as community assembly theory and ontogenetic habitat shifts. Nevertheless, significant knowledge gaps in our understanding of marine connectivity emerged from this work. The identified research priorities to improve understanding of ecological connectivity within temperate seascapes are listed in Table 4, organised by the themes of spatial and functional connectivity. Below, these are briefly discussed in relation to broad research topics that emerged from the data: 1) Biogeographical contextual influences on connectivity and ecosystem functioning in temperate coastal seascapes; 2) Data needs and technical challenges as barriers to enabling NbS policy and financing and; 3) Interactions across the triple planetary crisis anthropogenic pressures.

**Table 4:** Themed research priorities identified to improve understanding of ecological connectivity across the temperate coastal seascapes *(numbered order does not refer to prioritisation or ranking within themes)*

Spatial & Physical	Biodiversity & Trophic interactions
1) How does the configuration of habitats in the coastal temperate seascape influence the ability of species to move between them? 2) What is the impact of habitat size, distance etc. between biogenic/structured habitat on unstructured habitat communities? 3) What are existing barriers to coastal connectivity? 4) Colouring the ‘white ribbon’: How do we improve access to, and resolution of, data at the land/sea interface. 5) Consistent collation of nearshore data on a range of abiotic parameters (e.g., Bathymetry, wave climate, sediment type, light climate (PAR)). 6) Understanding the unanticipated effects of extreme events on connectivity. 7) How do soundscapes modify connectivity in the seascape? (and other anthropogenic stressors, e.g., artificial light at night; hormones & pharmaceuticals from wastewater) 8) High quality data on habitat extent, condition, and temporal and spatial changes.	1) Characterising “reference” trophic webs within an intact seascape (across multiple habitat configurations and settings). 2) How does habitat condition and context impact fish condition and survival? 3) How does habitat condition and context impact species composition, biodiversity, and biomass of coastal communities? 4) Understanding trophic links in saltmarshes and the bottom-up predator-prey interactions that supports fish production. 5) Measurements of local use of habitats by fish and their benefit on fish condition 6) Impact of fragmentation on fish growth, production, and survival (and utilisation/ movement between patches). 7) Methods for monitoring biotic habitat use in high tidal/ high energy/ low visibility areas. 8) Characterisation of the species community assemblages associated with *Ostrea edulis* habitat and the bottom-up impact of oyster reefs on trophic webs. 9) Effects of climate change on species interactions across the seascape and consequent impacts on ecosystem functioning.
**Genetics & Larval/Seed dispersal**	**Nutrients & Carbon**
1) Understanding the geographic scale of propagule connectivity (across/ between habitats) e.g., oyster larvae, kelp gametes 2) The impact of habitat extent & patchiness on connectivity of larval source populations. 3) Resolving the genetic population structure in remnant habitats of a) oysters, b) kelp, c) seagrass to inform existing connectivity and restoration. 4) Assess species composition of larval communities utilising different coastal habitats. 5) Observational data on biological behaviour, including larval swimming and diel vertical migration for accurate parameterization of dispersal models used in predicting connectivity patterns. 6) Potential for eDNA to elucidate larval transport, genetic population structure and connectivity. 7) Climate change & phenological mismatch impacts on habitat interactions	1) What are the key sources of primary production in the coastal seascape? 2) How do changes in water quality impact different habitats across the seascape; are their potential unintended consequences of restoration? 3) The effect of climate change on biotic composition and ranges, and the impacts of this on nutrient and carbon cycling. 4) Characterisation of movements/ flows of carbon and nutrients (N,S,P,Si) from landscape and between habitats across the seascape and implications for biogenic/ physical processing and inorganic/organic carbon storage. 5) Understanding seascape spatial configuration complexity and its relation to N and C sequestration and storage

Biogeographical contextual influences on connectivity and ecosystem functioning in temperate coastal seascapes. The results of our structured review identified a distinct geographical bias in the peer-reviewed literature regarding the role of connectivity in temperate coastal ecosystems (Fig. 4), with the majority of data derived from studies in specific regions; the Northern European Seas, Cold Temperate Northwest Atlantic and East Central Australian Shelf. The lack of data for South America is striking, although this may in part be due to the English language bias innate in our literature search. In addition, we have not taken a social science approach and investigated the records and stories of Indigenous peoples that would offer valuable insight not returned from our limited search terms. Globally, however, it is clear that the distribution of research effort is highly skewed towards the Atlantic coast of North America, Europe and Australia, perhaps reflecting temperate hotspots of marine restoration activity or the distribution of investment in ecological science.

A broad evidence-base for ecosystem services delivery across many habitats occupying the temperate coastal seascape exists at a global scale (Fig. 5), however ecosystem service assessment is lacking for many regional species (e.g., *O. edulis*^201^, see also Supplementary References 2). Better quantification of biophysical processes and rates (e.g., denitrification rates, CSS), combined with the drivers of variation in these assessments^289^, are needed to build confidence and provide contextual information for published values. These are essential to support monetary valuation and the development of high-quality marine natural capital finance markets^290,291^. There are still significant knowledge gaps related to the ecosystem services associated with specific habitats, in particular maerl and macro algae, and the cultural ecosystem services are far less quantified for all the habitats (Fig. 5). Our understanding of the impact of connectivity in relation to ecosystem services modulation is largely unknown across temperate seascape habitats (Fig. 7).

While many of the examples of ecological connectivity across the seascape, described above, (see also Fig. 6 & 7) are fairly well documented and supported, their quantitative application to specific regions remains a major knowledge gap for many parts of the world (Fig. 4). For example, it is increasingly apparent that many ecosystem functions show substantial spatial variation^186,292,293^ driven by regional differences in key drivers such as tidal range, rainfall patterns, and seascape configuration^184,185,294^. So, while it is clear that the range of factors described above all play roles in regulating the productivity of coastal seascapes for mobile fish and crustaceans, the specific details, such as threshold distances between habitat patches and interactions between tide range and habitat use, are unknown for many geographic locations. Quantifying the ranges over which contextual variables regulate geographic variation in seascape connectivity and function^72,177^ will help ensure that seascape connectivity is appropriately incorporated into coastal restoration efforts.

Many knowledge gaps are dependent on overcoming technical challenges or are required to remove barriers to the development of enabling policy and finance mechanisms (Table 4). For example, although the contribution of animal biomass to organic carbon standing stocks is thought to be significant, their functional roles in organic CSS are likely more important, but are not yet sufficiently understood, attributed or accounted for in carbon credit methodologies^295^. Similarly, whilst a seascape approach would allow a more holistic interpretation of what constitutes autochthonous organic carbon, to ensure management interventions maximise carbon sequestration and storage^273^, it remains important to understand the flow of organic carbon between different components of the seascape, however ascertaining the provenance of organic carbon presents an ongoing technical challenge. To date, bulk stable isotope analysis (e.g., δ^13^C and δ^15^N) is the most utilised technique, although resolving sources requires isotopically constrained systems^296^. Multi-proxy approaches can support inferences from bulk isotope analysis, for example, C/N ratios are an ideal supplementary tool given elemental analysis of carbon and nitrogen are usually completed alongside isotope analysis^297^ (Lamb et al., 2006). Other techniques, such as environmental DNA and compound-specific isotope analysis have also been utilised^298^ but ultimately it depends on the system in question whether these techniques are able to partially or fully resolve carbon provenance and the movement of carbon between habitats.

The three strands of the triple planetary crisis are interrelated, yet we know little about how these pressures interact to affect connectivity, ecosystem functioning and services in the temperate coastal seascape For example, whist the role of saltmarshes in filtering excess nutrients is recognised as an ecosystem service (Fig. 5 and sections above), nutrient enrichment has consequences for carbon cycling and the ability of saltmarsh to keep pace with sea-level rise (and thus has implications for the preservation of existing carbon stores). Excess nutrients have differing effects on above and below ground biomass, where the relative importance of differing mechanisms has not been fully elucidated and the net effect may depend on elevation^267,268^. Aboveground, increased stem density can boost sediment trapping, leading to enhanced accretion^268^, while belowground the development of weaker and less extensive root systems can leave the intertidal marsh vulnerable to erosion. Smothering by wrack (e.g., from *Ulva* blooms) can also disturb saltmarsh plant growth, an effect often limited to the low marsh^299^. In addition, nitrate pollution can increase litter decomposition by stimulating denitrification, altering the microbial community towards groups able to oxidise more complex organic matter that would otherwise be stored within the sediment^269^.

The importance of spatial and functional connectivity, and the capacity of coastal habitats (tidal flats, saltmarsh, oyster reefs, seagrass meadows) to withstand and assimilate increasing nutrient loads in temperate regions is largely unknown. The impact on the nutrient abatement capacity of co-located seagrass and saltmarsh and the effects of detrital and particulate transport on overall nutrient fluxes is also poorly understood. Similarly, the effect of seagrass patchiness, biodiversity, and health on nutrient dynamics has not been sufficiently explored. The lack of substantial areas of naturally-functioning native oyster reef and seagrass meadows is a major obstacle for demonstrating interactions. Current and future restoration projects operating at the seascape scale and involving multiple habitats provide living labs for assessing and delivering synergies whilst investigating these ecological connectivity research priorities.

### Recommendations to achieve seascape restoration of coastal habitats

Multi-habitat restoration enables synergies between neighbouring habitat patches and across habitat mosaics^55^ that may ultimately enhance ecological connectivity and ecosystem functioning at wider spatial scales and help to ensure that coastal ecosystems are resilient to future change. The seascape term and approach do not seek to replace existing management approaches such as ecosystem-based management and Integrated Coastal Zone Management, instead, it informs these approaches by providing operationally relevant ecological information on the consequences of landscape and seascape structure on functions such as connectivity, integrity and resilience that are central to ecosystem-based management^300^. The evidence presented above is assimilated into a list of recommendations to deliver seascape restoration below:

1. Utilise networks to facilitate knowledge exchange, develop skills and create guidance to deliver multiple habitat restoration.
  Restoration of multiple habitats concurrently or sequentially is necessary to restore functionality and connectivity across the mosaic of habitats in our temperate coastal seascapes. Adopting a multi-habitat approach that spans the land-to-sea interface, facilitates the consideration of biophysical and social contexts, the spatial patterns of habitat across the landscape or seascape and the influences of connectivity^129^. However, to achieve this, we need effective platforms for knowledge exchange and capacity building across multiple sectors and disciplines.
  Marine habitat restoration is both sufficiently complex, and recent, that it has been necessary to focus on achieving success with one habitat at a time, e.g., oyster reef^301–303^, kelp forests^304^, mussel beds^305^, seagrass^306^ and saltmarsh^307^. *With growing understanding of, and success in, restoring individual habitats, now is the time to build on our single habitat expertise and create effective networks to deliver seascape restoration*.
2. Integrate seascape ecology and an understanding of connectivity into the restoration planning stages of multi-habitat seascape restoration projects.
  Seascape ecology provides descriptions of the importance of spatial patterns (e.g., patch area, perimeter: area ratio, connectivity, mean patch fractal dimension) among marine habitats in understanding the seascape composition and configuration^66^. This provides the theoretical basis for designing restoration on a seascape scale including the important habitat types in each location. A seascape ecology approach enables a consideration of the influence of seascape structure on the local or regional coastal restoration potential and can inform the prioritisation and scaling-up of restoration efforts. Undesirable effects of connectivity also need to be considered, and may include spread of pathogens, pollutants and invasive species, therefore, proper assessment of conservation benefits and risks must be taken with consideration of spatial scales^61^ and appropriate biosecurity measures^308^*. How interconnections across the seascape influence local site conditions and the potential restoration suitability, is particularly important to consider when scaling up coastal NbS*^289^.
3. Develop restoration and site suitability models for multiple coastal habitats to aid restoration planning.
  Site suitability (the potential for a selected site to support the desired habitat) is arguably the most fundamental consideration in ecological restoration planning. Restoration suitability modelling for planning purposes takes that concept a step further by including feasibility criteria, including the practicalities involved in restoration; access to the water, loading facilities for the equipment required for restoration, and the social and management environment of different locations^291,309^. An example of this is for the Reef Builder programme in Australia, where a need to implement many shellfish restoration projects in parallel to deliver a large and broad scale restoration programme of works required a standardised approach to guide restoration site selection that accounted for the shifting seascape mosaic and prioritised efforts based on the best available present-day data^309^. *Restoration suitability models need to be more widely developed to include relevant biological, ecological, logistical, and social parameters*.
4. Develop decision-making tools to inform the order of habitat restoration when planning seascape restoration.
  Decision making tools that allow for the causal chains that can occur due to interactions between restoration measures and the seascape context are needed to inform restoration planning. Here, logic models or theories of change can be used when planning a restoration project to develop the causal links that will help articulate the expected or conceptual model for a seascape restoration progression. For example, taking oyster and seagrass habitats: considerations of the existing seascape context (water quality) and connectivity will inform your approach. The first action may be to remove water quality pressures (often considered passive restoration, however, considered here part of the seascape ecosystem restoration spectrum, Fig. 1), otherwise, excess sediment delivery from land and/or by dredging may smother habitat restoration attempts. Eutrophication may be additionally bioremediated by first implementing oyster reef restoration. These actions can be used to improve water quality to increase likelihood of successful seagrass restoration, or enable natural expansion without active intervention. This may lead to increased CSS that can be leveraged via carbon markets to finance further restoration. This approach can be used to develop an expected option set or appraisal of the types of connectivity that will likely arise or benefit a project because of the restoration or protection of habitats. *Logic models can then be used to guide Climate-smart marine spatial planning (MSP) and factor restoration into the wider consideration of a functioning seascape and its human and natural values*.
5. Seascape restoration needs to be a component of climate-smart marine spatial planning.
  Climate-smart MSP is a tool to manage ocean uses coherently and to ensure that human activities take place in an efficient, safe, and sustainable way^310^. Incorporating seascape restoration into climate-smart MSP will ensure that areas critical for ecosystem services and biodiversity are effectively protected and restored. By prioritising connectivity and associated ecosystem functionality, climate-smart marine spatial planning can help maintain the integrity and resilience of marine ecosystems in the face of a changing climate^310,311^. Restoration of coastal ecosystems is an activity that needs to be considered and planned for alongside management (e.g., aquaculture, farming and extractive activities (e.g., fishing, water, substrate)) that take place across the seascape and beyond into the surrounding catchment. Marine spatial plans that prioritize the need to restore seascape connectivity to ensure the flow of species, nutrients and energy across multiple habitats will benefit from the recovery of key ecosystem processes, delivery of sustainable benefits across a range of user groups and maximize seascape resilience in a climate change. This is critical to ensure considerations of ecological connectivity between the seascape and the landscape, and within habitats across the seascape mosaic to enable management of resource use that will allow for such connections. *Site selection and scaling could likely be improved by approaching seascape restoration as nature-based solutions within a larger-scale climate-smart MSP framework to help overcome short policymaking timeframes and ensure that ocean health is lasting*^312,313^.
6. Marine restoration governance needs to mature to deliver projects at scale.
  A seascape approach will, by necessity, be interdisciplinary, require multiple partners and involve a range of stakeholders. Developing appropriate governance processes that are inclusive of all stakeholders, empowering and transparent as set out by the SER and IUCN Global standards for Nature-based solutions^314,315^ is essential to successful NbS. This approach drives momentum and collaborations, increases knowledge exchange and can generate social acceptance, all of which supports a transition of restoration at large scales. However, within this approach, the licencing and regulatory environment to enable marine habitat restoration needs to develop in parallel with advances in restoration ecology and practice to allow for connectivity between multiple habitats to be considered. For example, seagrass and shellfish habitats are two critical coastal habitats in the temperate zone for which proximity can influence connectivity, yet the historic recognition of the importance, and threatened status, of seagrass has resulted in protections that make it difficult, if not impossible, to consider restoring them together in the same location^309^. *This emphasises the need for practitioners, regulators and scientists to collaborate to ensure governance recognises advances in restoration science and practice, to ensure enabling regulatory and licensing frameworks are established for seascape restoration at scale*.
7. Align monitoring frameworks with research agendas and policy goals to increase confidence in outcomes and support nature-positive finance markets.
  We need to learn while implementing seascape restoration. There is an opportunity for researchers to co-design experimental studies with restoration practitioners and communities to understand structure-function relationships that will lead to better multi-habitat restoration outcomes, particularly as the data required to validate this increasingly complex endeavour will be considerable. Robust quantification of ecosystem services delivered by habitats created by seascape restoration can then be leveraged into cost-benefit analyses and blue finance models to further scale up efforts and assess progress against goals^316^. Developing coherent monitoring approaches that include metrics that also assess connectivity, can be a powerful approach to provide data for research priorities and help address the knowledge gaps discussed above. In Europe, restoration monitoring could be strengthened if delivered under existing frameworks, such as the Marine Strategic Framework Directive monitoring programme. The use of integrated (inclusive of ecological, social and economic aspects) monitoring, evaluation and learning frameworks^317,318^ can facilitate adaptive approaches and management, as we are informed by evidence from restoration outcomes. *Given the urgency of the need to act to meet our global targets, it is strongly recommended that society takes a proactive and iterative approach to achieve restoration at scale*.

### Policy pathways to achieve seascape recovery

As evidenced throughout this paper, ecological connectivity across the temperate seascape underpins the delivery of critical functionality, ecosystem services and resilience. However, overlapping objectives within various biodiversity and climate policies create a complex and inefficient landscape for achieving nature recovery^319^. There remains ample opportunity to develop policy pathways further to ensure consistent ecosystem restoration targets and delivery strategies are underpinned by seascape connectivity. A summary of global and regional policy instruments underpinned through seascape connectivity are listed in Table 5. Given the significance and evidence of ecological connectivity presented here, we highlight two policy opportunities and suggested actions to deliver seascape benefits from restoration at scale:

**Table 5:** Global and example regional policy instruments that are underpinned through seascape connectivity

Those unshaded explicitly refer to connectivity, those shaded in grey are impacted by connectivity but do not explicitly use the term.

1. Transition away from feature-based designations to assessments for protection and management, which account for connectivity and the delivery of ecosystem services.
  The emerging global policy landscape recognises that the nature protection provided through previous treaties is not having the intended outcome of healthy seas and land. In the UK, a recent assessment estimated only 44% of English MPA protected features are in a favourable condition^320,321^ and 11 of the 15 indicators did not meet Good Environmental Status (GES) for UK marine ecosystems^322^, demonstrating that existing management measures have had limited success. Similarly, MPAs in Europe are mostly designated and monitored using a feature-based approach, wherein protection of listed species and habitats is prioritized prioritised^323,324^. This approach overlooks the seascape connectivity that supports those protected features and therefore the wider protected area^325^. For example, the removal of bottom-towed fishing from a protected seagrass meadow will limit impacts in the delineated seagrass feature but allow it to otherwise continue in the MPA, despite damaging critical connecting habitats (e.g., soft sediments) and creating additional threats (e.g., smothering from nearby sediment disturbance)^326,327^. In addition, the baseline on which feature-based approaches are managed is often a highly degraded baseline, not accounting for what is required to deliver functionality or resilience to future climate scenarios (e.g., *O. edulis* habitat descriptions^18,328^).

Recommended actions:

- Recognise the potential impact of **degraded baselines** on nature protection targets, utilising historical and scientific data (e.g., ecological connectivity and climate forecasting) to inform restoration targets where ecosystems have collapsed^30^;
- Construct appropriate targets for nature protections that account for what is required to restore ecosystem **functionality** and deliver **resilience** (via ecosystem services) for future climate scenarios;
- Implement **whole-site** management approaches that consider activities across **ecosystems** in the seascape, not just across individual protected **features**, to ensure effective protection of the MPA network^325^.

2. Seascape connectivity offers an opportunity to integrate policy frameworks across climate and biodiversity agendas.
  Marine NbS are increasingly recognised for their capacity to deliver for both climate and biodiversity commitments. Under the Paris Agreement, 97 countries included coastal and marine NbS such as habitat restoration, in their Nationally Determined Contributions (NDCs)^329,330^, aligning UNFCCC climate-related efforts with the CBD agenda. The first global stocktake noted *‘the urgent need to address… the interlinked global crises of climate change and biodiversity loss…as well as the vital importance of protecting, conserving, restoring and sustainably using nature and ecosystems for effective and sustainable climate action’*^330^. In addition, under Article 6 of the CBD, parties are explicitly required to develop National Biodiversity Strategies and Action Plans (NBSAPs) and require parties to: ‘*Integrate, as far as possible and as appropriate, the conservation and sustainable use of biological diversity into relevant sectoral or cross-sectoral plans, programmes and policies’*. This is reflected in the recommendation for UK national climate and biodiversity strategies to be spatially explicit to support implementation of the other^331^, as reflected in Recommendation Five above. The EU Restoration law also recognises that ‘*securing biodiverse ecosystems and tackling climate change are intrinsically linked’*.
  Seascape connectivity evidence highlights opportunities to streamline monitoring across climate and biodiversity policy frameworks when implementing and reporting nature recovery and NbS. Building on expertise and knowledge within terrestrial environment conservation^332,333^, it is timely to develop a suite of metrics to assess connectivity and associated ecosystem functionality and service delivery at seascape scales. This monitoring, if leveraged effectively, could be vital in underpinning the development of finance markets to meet the considerable funding gap required to achieve our restoration targets.

Recommended Actions:

- **Monitoring** and **reporting** to be streamlined across global policies linked to ecosystem restoration, to provide consistent data that facilitates efforts towards effective global restoration initiatives^319,334^
- Identify **priority seascape restoration metrics** to be integrated into global and national monitoring frameworks, such as for GBF target 2.
- Development of **high-quality natural capital markets** to finance nature restoration^290^ (e.g., Finance Earth, 2024), utilising metrics used in global monitoring frameworks.

## Discussion

Global and national policies and commitments to protect and restore marine habitats and species are numerous and continually emerging. However, despite underpinning successful delivery (see Table 5), seascape connectivity has not historically been considered within these frameworks and ambitions. New international frameworks and their domestic implementations are beginning to explicitly mention ecosystem quality, function and interconnectedness as essential for nature recovery and societal benefits, and legally binding targets must be delivered at a national level by all signatory Parties. The evidence presented in this paper highlights the relevance and necessity of seascape connectivity in achieving various global and national strategies and policies.

The Kunming-Montreal Global Biodiversity Framework^335^ adopted four outcome-oriented goals, two of which address connectivity and nature restoration: Goal A ‘*protect and restore*‘ and Goal B ‘*to prosper*’. To achieve the goals, the GBF includes 23 action-focused targets for 2030. Coastal ecosystem recovery and connectivity is fundamental to achieving Targets 1-4. Target 2 explicitly commits countries to have ‘*30% of all degraded ecosystems under effective restoration by 2030 to enhance ecosystem functions, ecological integrity and connectivity’*. Target 2 defines ‘*high ecological integrity*’ as an area with composition, structure, function and ecological process close to that of a natural ecosystem, and that connectivity ensures the maintenance of natural species habitats. A further demonstration of frameworks examining connectivity within policy implementation is the Convention of Migratory Species^336^, a global convention dedicated to conserving migratory species, their habitats and migration routes. The CMS has 133 national signatories and covers many species in temperate coastal zones, particularly seabirds. At the CMS COP14 in Uzbekistan, 2024, parties passed a resolution that, for the first time globally, recognises the role that seagrasses play in supporting migratory species and places the onus upon signatory states to ensure their conservation.

The connectivity science of temperate coastal ecosystem similarly underpins the implementation of several regional policies, including the EU Nature Restoration Law adopted on 17 June 2024, which sets legally binding targets for the EU member states to restore at least 20% of the EU’s land and sea areas by 2030 and all ecosystems in need of restoration by 2050. The EU Restoration law aims to achieve the EU’s climate and biodiversity objectives as well as meet the GBF goals and targets. As with the GBF, member states have two years from adoption to submit National Restoration Plans to the commission, detailing measures in place by 2030 to restore at least 30% of the habitat types listed in Annex I and II of the Habitats Directive. The EU Restoration law, aligned with the focus on connectivity in Target 2 of the GBF, also states the need to improve connectivity of habitat types listed in its Annex I (Article 4, 10), and of the coastal (freshwater & terrestrial) habitats of the species listed in Annexes II, IV and V (Article 5, 7) and improve ecological coherence (Article 5, 8) between the habitats, particularly wetlands (Article 60) including those that span borders (Article 65), to maximise efficiency of restoration efforts (Article 33), to achieve sufficient habitat quality and quantity (Article 5, 5 and Article 4, 7) and support thriving and climate resilient species populations (Article 14, 2b). Annex VII also lists improving connectivity across habitats as a restoration activity to enable species genetic exchange, migration and climate change adaptation (Article 22).

In addition, although not in itself legally binding, the recent International Tribunal for the Law of the Sea (ITLOS) advisory opinion^337^ found that under the United Nations Convention on the Law of the Sea (UNCLOS) GHG emissions are a marine pollutant and states are legally obliged to protect and preserve the marine environment from its deleterious effects, including restoring degraded habitats and ecosystems. This provides clarity on the actions needed under due diligence, and calls for global and regional cooperation in meeting member states obligations under UNCLOS, including acting on the best available science.

The way we think about coastal seascapes affects the way in which they are managed and restored. Restoring ecological connectivity supports ecosystem functioning and biodiversity by facilitating the flow of matter, energy and organisms between habitats (e.g., ontogenetic movements between important feeding or nursing areas)^42^. Now, halfway through the UN Decade of Ecosystem Restoration, it has never been more important to develop a seascape approach to restoration that considers the wider habitat mosaic and accounts for the connectivity between habitats, with the overarching aim of equipping managers and practitioners with a science-based foundation from which they can build sustainable coastal seascape restoration strategies^338^. To support this action this paper provides clear definitions, scientific evidence and practical recommendations for managers and practitioners to achieve restoration at scale in temperate coastal ecosystems.

Addressing the policy opportunities outlined here will also help provide the pathway to ensuring the successful implementation of critical global and national policies and support the ambitious targets of the Decade on Ecosystem Restoration (2021–2030) to revive ocean ecosystems and establish best practices for seascape recovery, management and assessment^47^ (United Nations Environment Programme, 2019), and address the ambitions and challenges outlined in the UN Decade of Ocean Science for Sustainability (UNESCO-IOC, 2021).

## Conclusion

**Fig. 8:**
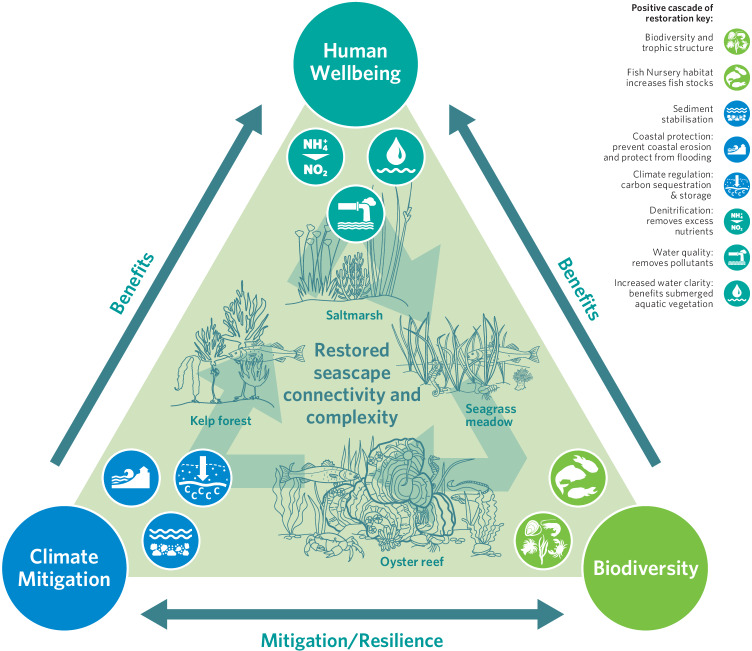
Conceptual diagram of how ecosystem services from a restored and connected seascape underpins the interrelationships between climate mitigation, biodiversity and human wellbeing.

Anthropogenic stressors have diminished nearly all coastal marine habitats, and globally almost all marine biomes are impacted by overfishing, pollution and climate change. To reverse the global trend of biodiversity loss we need urgent, bold and effective restoration action. The evidence presented here is striking and clear; to achieve ‘*a healthy and resilient ocean where marine ecosystems are understood, protected, restored and managed’* and to deliver on climate mitigation and sustainability goals, we need to restore at seascape scales, reconnecting the matrix of coastal marine habitats to restore the resilience and functionality of the temperate seascape on which human wellbeing depends (Fig. 8).

## Methods

### Expert opinion: Symposium & technical workshop on seascape ecological connectivity

The symposium on ‘Ecological connectivity across temperate coastal habitats’ was held at the ZSL, UK, on 22nd and 23rd November 2022. The Symposium was attended by 150 delegates, bringing together scientists, regulators, policy makers and practitioners with expertise across key coastal habitats to facilitate knowledge exchange, review our understanding of ecological connectivity and interactions across the temperate coastal seascape, and finally, undertake priority setting to enable the application of seascape connectivity in restoration policy and practice. On the 24th November, 41 of the symposium delegates participated in a one-day technical workshop, by invitation, to conduct a comprehensive state of knowledge analysis on connectivity and interactions across temperate coastal seascapes.

Initial data were compiled from expert discussion collated during the symposium. Day one featured presentations and panel sessions on: international context for seascape restoration, historical ecology and current knowledge of temperate marine habitats, integrated habitat restoration and seascape connectivity. A chaired discussion on mechanisms and evidence of connectivity followed, during which delegates contributed examples and evidence of functional connectivity and mechanisms of connectivity (via Post-it notes and subsequently collated into a live document) to inform the technical workshop. Day two featured presentations and panel sessions on: the science of connectivity, decision making in habitat restoration, coastal restoration and the future. A chaired discussion gathered delegate input on the importance of integrating seascape restoration into policy and practice, addressing the following questions: *Why do you consider it important to take a seascape approach to restoration? Which policies would benefit from having a better understanding of connectivity and why? What are the main barriers to operationalising seascape restoration?* All answers were captured via an open notice board.

The technical workshop on seascape connectivity began by gathering expert opinion to construct a definition of the temperate coastal seascape and select the habitats to incorporate within the structured review. Attendees then reviewed the types of marine ecological connectivity and facilitating mechanisms collated during the symposium. Breakout groups focused on specific subtopics: structural connectivity (physical habitat interactions) or aspects of functional connectivity; movement of organisms, (behavioural and trophic), gametes and larvae (genetic and population) or non-living matter (nutrients and carbon). Each group reviewed the list of types and mechanisms of connectivity, providing evidence, examples, and supporting data. Subsequently, knowledge gaps were identified, including those related to potential mechanisms or types of connectivity for which data are lacking in temperate coastal systems. Summary sheets of priority knowledge gaps and methods to address these were drafted. Attendees then discussed evidence for the scales over which the collated examples of connectivity operated. The workshop ended with a plenary session on priority setting, where the following key questions were explored: *Why is ecological connectivity important in the temperate coastal seascape? What does seascape connectivity deliver for societal goals?* The plenary concluded with a review of relevant policies. Finally, attendees were asked to add ecosystem service categories to the previously identified links between connectivity mechanisms and the ability to deliver SDGs and climate goals (captured on padlet).

### Structured review

Based on the expert opinion gathered during the symposium and workshop, a structured literature review was carried out during 2022/23 to create a database on the published evidence of connectivity across the temperate seascape (full database available, see data availability statement). The initial literature search was conducted through Web of Science (WoS) (01/11/2022) using the following search terms: (connectivity OR seascape* OR meta-ecosystem* OR habitat-mosaic* OR mosaic-habitat* OR (habitat AND mosaic*)) AND (coastal OR marine OR estuar* OR ocean*) AND (((saltmarsh* OR (tidal AND marsh*)) AND (seagrass* OR macroalga* OR seaweed* OR macrophyte* OR kelp* OR oyster* OR mudflat* OR ((tidal OR intertidal) AND flat*))) OR (seagrass* AND (saltmarsh* OR (tidal AND marsh*) OR macroalga* OR seaweed* OR macrophyte* OR kelp* OR oyster* OR mudflat* OR ((tidal OR intertidal) AND flat*)))), seagrass* AND (saltmarsh* OR marsh*) AND nutr* AND nitr* AND phosph*AND temperate. This resulted in 187 papers, including 15 reviews. Cross referenced papers were subsequently explored and added to the database of 215 peer-reviewed publications (09/12/2022).

Publications were categorised manually by type^339^; flows (physical, carbon, nutrient, biodiversity), location (distinct location and biogeographic region), habitats included (seagrass, saltmarsh, oyster reef, coral reef, mangrove, unvegetated sediment, seaweed/rock, rhodolith, beach/dune, offshore, terrestrial), taxonomic focus, conservation focus, generation of empirical data, use of numerical model, cultural ecosystem service focus. Biogeographic region was assigned using the Marine Ecosystems of the World (MEOW)^340^, which consists of 12 realms (Arctic; Temperate Northern Atlantic; Temperate Northern Pacific; Tropical Atlantic; Western Indo-Pacific; Central Indo-Pacific; Eastern Indo-Pacific; Tropical Eastern Pacific; Temperate South America; Temperate Southern Africa; Temperate Australasia; Southern Ocean) and nested 62 provinces. For this paper, the tropical regions were excluded in further analysis. Each paper was assigned a value determined by the relevance multiplied by robustness. This value was used to prioritise focus on relevant papers, to identify areas of evidence gaps^339^ and identify problems with the search terms. In addition, expert knowledge was used to add scientific papers not identified in WoS or covered by the search terms to the database, via the outputs from the workshop and during the writing of the manuscript (304 publications).

To assess the evidence of the role of connectivity on ecosystem service delivery within the seascape, the database was reviewed further to identify publications that measured or inferred the impact of connectivity between habitats on the provision of an ecosystem service. This subset of publications was expanded by expert knowledge from contributing authors to capture relevant publications that were not identified in the initial structured literature review. The connected habitat types, where the change in ecosystem service was measured or inferred, and the direction of any change in ecosystem service provision were extracted. The strength of evidence for connectivity between the habitats affecting ecosystem service provision was assigned a weighting based on the nature of the evidence presented (inferred = 1, indirect evidence of a process occurring = 2, quantitative = 3) and the number of publications in each case. This allowed the data gaps and the strength of evidence in each case to be visualised by plotting the sum of the evidence weighting for each ecosystem service-habitat pairing.

## Supplementary information

Supplementary Information

## Data Availability

All data are available in the supplementary materials and in the Figshare database titled “Preston et al. 2025 Supplementary Table: Marine Temperate Habitat connectivity evidence from structured review” available at 10.6084/m9.figshare.28768307.
