# Supplementary material for: Seascape connectivity: evidence, knowledge gaps and implications for temperate coastal ecosystem restoration practice and policy
Source: NPJ Ocean Sustain. 2025 Jun 12;4(1):33. doi: 10.1038/s44183-025-00128-3 (PMC12162346; doi:10.1038/s44183-025-00128-3)
Supplement: Supplementary file 1 — Supplementary Information [file 44183_2025_128_MOESM1_ESM.pdf]

Supplementary References 1.

References for Table 1: listed by habitats

OSPAR List of threatened and/or declining habitats: https://www.ospar.org/work-areas/bdc/species-habitats/list-of-threatened-declining-species-habitats/habitats. *Accessed March 12th 2025*.

Saltmarsh

Adam, P. Saltmarshes in a time of change. *Environmental Conservation*. **29**(1), 39-61 (2022).

Campbell, A. D., Fatoyinbo, L., Goldberg, L. & Lagomasino, D. Global hotspots of salt marsh change and carbon emissions. *Nature*. **612**, 701–706 (2022).

Crooks, S., Herr, D., Tamelander, J., Laffoley, D. & Vandever, J. Mitigating climate change through restoration and management of coastal wetlands and near-shore marine ecosystems : challenges and opportunities. *Environment Department Papers.* **121** (2011).

Duarte, C., Dennison, W., Orth, R.W. & Carruthers, T.B. The Charisma of Coastal Ecosystems: Addressing the Imbalance. *Estuaries and Coasts.* **31**(2), 233-238 (2008).

Gedan, K. B., Silliman, B. R., & Bertness, M. D. Centuries of human-driven change in salt marsh ecosystems. *Annu. Rev. Mar. Sci.* **1**(1), 117-141 (2009).

Hudson, R., Kenworthy, J. & Best, M. *Saltmarsh Restoration Handbook: UK and Ireland.* (Environment Agency, 2021).

Maxwell, T.L. et al. Soil carbon in the world's tidal marshes. *Nat. Commun.* **15**, 10265; https://doi.org/10.1038/s41467-024-54572-9 (2024).

Mcowen, C. J. et al. A global map of saltmarshes (V6.1). *Biodivers. Data J*. **5**, e11764; 10.3897/BDJ.5.e11764 (2017).

Mcowen, C. J. et al. A global map of saltmarshes (V6.1) data sets. *Ocean Data Viewer*; https://doi.org/10.34892/07vk-ws51 (2017).

Pétillon, J. et al. Top ten priorities for global saltmarsh restoration, conservation and ecosystem service research. *Science of The Total Environment*, **898**, 165544; https://doi.org/10.1016/j.scitotenv.2023.165544 (2023).

Worthington, T. A. et al. The distribution of global tidal marshes from Earth observation data. *Global Ecology and Biogeography*, **33**, e13852; https://doi.org/10.1111/geb.13852 (2024).

Zhang, X. et al. GWL_FCS30: A global 30 m wetland map with a fine classification system using multi-sourced and time-series remote sensing imagery in 2020. *Earth System Science Data*, **15**(1), 265–293 (2023).

Seagrass Meadow

Dunic, J.C., Brown, C. J., Connolly, R. M., Truschwell, M. P. & Côté, I. M. Long-term declines and recovery of meadow area across the world's seagrass bioregions. *Global Change Biology*, **27**(17), 4096-4109 (2021).

Fikiris, E. et al. Predictive Mapping of Mediterranean Seagrasses - Exploring the Influence of Seafloor Light and Wave Energy on Their Fine-Scale Spatial Variability. *Remote Sens*. **15**, 2943; doi:10.3390/rs15112943 (2023).

Gamble, C. et al. *Seagrass Restoration Handbook.* (Zoological Society of London, 2021).

Lee, K-S., Park, S. R. & Kim, Y. K. Effects of irradiance, temperature, and nutrients on growth dynamics of seagrasses: A review. *J. Exp. Mar. Biol. Ecol*. **350**, 144–175; doi:10.1016/j.jembe.2007.06.016 (2007).

McKenzie, L. J. et al. The global distribution of seagrass meadows. *Environ. Res. Lett*. **15**, 074041 (2020).

Orth et al. A Global Crisis for Seagrass Ecosystems. *BioScience*. **56**, 987-996 (2006).

Short, F. T. *World Atlas of Seagrasses* (University of California Press, 2003).

Short, F., Carruthers, T., Dennison, W. & Waycott, M. Global seagrass distribution and diversity: A bioregional model. *J. Exp. Mar. Biol. Ecol*. **350**, 3–20; doi:10.1016/j.jembe.2007.06.012 (2007).

Turschwell, M. P. et al. Anthropogenic pressures and life history predict trajectories of seagrass meadow extent at a global scale. *PNAS*, **118** (45), e2110802118 (2021).

Waycott, M. et al. Accelerating loss of seagrasses across the globe threatens coastal ecosystems, *PNAS*. **106**(30), 12377-12381 (2009).

Macroalgae seaweed (excluding kelp)

Duarte, C. M. et al. Global estimates of the extent and production of macroalgal forests. *Global Ecology and Biogeography*. **31**(7), 1422-1439 (2022).

Edworthy, C., Steyn, P-P., & James, N.C. The role of macroalgal habitats as ocean acidification refugia within coastal seascapes. *Cambridge Prisms: Coastal Futures*. **1**(e22), 1–10; https://doi.org/10.1017/cft.2023.9 (2023).

Fulton, C. J. et al. Macroalgal meadow habitats support fish and fisheries in diverse tropical seascapes. *Fish and Fisheries*. **21**(4), 700-717 (2020).

Joniver, C. F. et al. The global problem of nuisance macroalgal blooms and pathways to its use in the circular economy. *Algal Research*. **58**, 102407 (2021).

Middelboe, A. L., & Hansen, P. J. High pH in shallow-water macroalgal habitats. *Marine Ecology Progress Series.* **338**, 107-117 (2007).

O'Brien, J. M. & Scheibling, R. E. Turf wars: competition between foundation and turf-forming species on temperate and tropical reeds and its role in regime shifts. *Marine Ecology Progress Series*. **590**, 1-17 (2018).

Spalding, H. L. et al. Macroalgae in *Mesophotic coral ecosystems* (ed. Loya, Y., Puglise, K.A. & Bridge, T.C.L.) 507-536 (Springer, 2019).

Walker, D. I., & Kendrick, G. A. Threats to macroalgal diversity: marine habitat destruction and fragmentation, pollution and introduced species. *Botanica Marina.* **41**, 105-112 (1998).

__Vermetid reefs__

Antonioli, F., Chemello, R., Improta, S. & Riggio, S. Dendropoma lower intertidal reef formations and their palaeoclimatological significance, NW Sicily. *Mar. Geol.* **161**, 155–170 (1999).

Azzopardi, L. & Schembri, P. J. Vermetid crusts from the Maltese Islands (Central Mediterranean). *Mar. Life.* **7**, 7–16 (1997).

Badreddine, A., Milazzo, M., Saab, M. A.-A., Bitar, G. & Mangialajo, L. Threatened biogenic formations of the Mediterranean: Current status and assessment of the vermetid reefs along the Lebanese coastline (Levant basin). *Ocean Coast. Manag*. **169**, 137–146 (2019).

Bisanti, L., Visconti, G., Scotti, G., Chemello, R. Signals of loss: Local collapse of neglected vermetid reefs in the western Mediterranean Sea. *Marine Pollution Bulletin*, **185**, Part B (2022).

Calvo, M., Templado, J. & Penchaszadeh, P. E. Reproductive biology of the gregarious Mediterranean vermetid gastropod Dendropoma petraeum. *J. Mar. Biol. Assoc. U. K.* **78**, 525–549 (1998).

Chemello, R. Marine bioconstructions in the Mediterranean Sea. A state-of-the-art on the vermetid reef. *Biol. Mar. Mediterr*. **16**, 2–18 (2009).

Chemello, R., Dieli, T. & Antonioli, F. Il ruolo dei "reef" a Molluschi vermetidi nella valutazione della biodiversità in *Mare e cambiamenti globali.* 105-118 (Quaderni ICRAM, 2000).

Chemello, R. & Silenzi, S. Vermetid reefs in the Mediterranean Sea as archives of sealevel and surface temperature changes. *Chem. Ecol*. **27**, 121–127 (2011).

La Marca, E. C. et al. Canopy-forming algae improve the colonization success of the reef-builder Dendropoma cristatum (Biondi 1859) on artificial substrates. *Front. Mar. Sci*. **10** (2024).

La Marca, E. C., Catania, V., Quatrini, P., Milazzo, M. & Chemello, R. Settlement performance of the Mediterranean reef-builders Dendropoma cristatum (Biondi 1859) in response to natural bacterial films. *Mar. Environ. Res*. **137**, 149–157 (2018).

Milazzo, M., Fine, M., La Marca, E. C., Alessi, C., and Chemello, R. Drawing the line at Neglected Marine Ecosystems: Ecology of Vermetid reefs in a changing ocean in *Marine Animal Forests* (ed. Rossi, S.) 1–23 (Springer eBooks, 2016). doi:10.1007/978-3-319-17001-5_9-1.

Picone, F., Sottile, G., Fazio, C., Chemello, R. The neglected status of the vermetid reefs in the Mediterranean Sea: a systematic map. Ecological Indicators, **143** (2022).

__Kelp Forest__

Eger, A. M. *et al.* The value of ecosystem services in global marine kelp forests. *Nature Communications.* **14** (2023).

Eger, A. M., Layton, C., McHugh, T. A., Gleason, M., and Eddy, N. *Kelp Restoration Guidebook: Lessons Learned from Kelp Projects Around the World* (ed. Caselle, J. & DeAngelis, B.) (The Nature Conservancy, 2022).

Jayathilake, D.R.M. & Costello, M.J. Version 2 of the world map of laminarian kelp benefits from more Arctic data and makes it the largest marine biome. *Biological Conservation*. **257**, 109099; https://doi.org/10.1016/j.biocon.2021.109099 (2021).

Krumhansl, K. A. et al. Global patterns of kelp forest change over the past half-century. *Proceedings of the National Academy of Sciences*. **113**(48), 13785-13790 (2016).

Smale, D. A. Impacts of ocean warming on kelp forest ecosystems. *New Phytologist*. **225**(4), 1447-1454 (2020).

Steneck, R. S. et al. Kelp forest ecosystems: biodiversity, stability, resilience and future. *Environmental Conservation.* **29**(4), 436-459 (2002).

Teagle, H., Hawkins, S. J., Moore, P. J., & Smale, D. A. The role of kelp species as biogenic habitat formers in coastal marine ecosystems. *J. Exp. Mar. Biol. and Ecol.* **492**, 81-98 (2017).

Wernberg, T., Krumhansl, K., Filbee-Dexter, K., & Pedersen, M. F. *Status and trends for the world's kelp forests in World seas: An environmental evaluation* (ed. Sheppard, C.) 57-78 (Academic Press, 2019).

Rhodolith (Maerl) Beds

De Araújo Costa, D. *et al.* An Overview of Rhodoliths: Ecological importance and conservation emergency. *Life.* **13**, 1556 (2023).

Nelson, W. A., & Adey, W. H. The Nature of Rhodoliths: Their Role as Habitat Formers. *Proceedings of the Seventh International Coral Reef Symposium*. **1**, 675-682 (1999).

Otero-Ferrer, F., Cosme, M., Tuya, F., Espino, F. & Haroun, R. Effect of depth and seasonality on the functioning of rhodolith seabeds. *Estuarine, Coastal and Shelf Science.* **235**, 106579 (2020).

Riosmena-Rodriguez, R., & Nelson, W. A. *The Rhodolith/Coralline Algae Biota of the World: Diversity, Ecology, and Conservation* (Springer, 2017).

Biogenic bivalve reefs

Baden, S., Hernroth, B., and Lindahl, O. Declining Populations of *Mytilus spp.* in North Atlantic Coastal Waters - A Swedish Perspective. *Journal of Shellfish Research*. **40**(2), 269 - 296 (2021).

Chan, S. S. et al. Increased biodiversity associated with abandoned benthic oyster farms highlight ecosystem benefits of both oyster reefs and traditional aquaculture. *Front. Mar. Sci.* **9**, 862548 (2022).

Edgar, G. J. *Australian Marine Life.* (New Holland Publishers, 2012).

Fariñas-Franco, J.M. et al. Marine Strategy Framework Directive Indicators for Biogenic Reefs formed by Modiolus modiolus, Mytilus edulis and Sabellaria spinulosa Part 1: Defining and validating the indicators. *JNCC Report No. 523*. (Joint Nature Conservation Committee, 2014).

Fitzsimons, J. A. et al. Restoring shellfish reefs: Global guidelines for practitioners and scientists. *Conserv. Sci. Pract.* **2**, e198 (2020).

Gubbay, S. et al. European red list of habitats, 1: Marine Habitats. 52 (Publications Office of the European Union, 2016).

Kellogg, M. L., Cornwell, J. C., Owens, M. S. & Paynter, K. T. Denitrification and nutrient assimilation on a restored oyster reef. *Mar. Ecol. Prog. Ser.* **480**, 1–19 (2013).

Lam, K. & Morton, B. The oysters of Hong Kong (Bivalvia: Ostreidae and Gryphaeidae). *Raffles Bull. Zool.* **52**, 11–28 (2004).

Lau, S. C., Thomas, M., Hancock, B. & Russell, B. D. Restoration potential of Asian oysters on heavily developed coastlines. *Restor. Ecol.* **28**, 1643–1653 (2020).

OSPAR Commission. Intertidal Mytilus edulis beds on mixed and sandy sediments. *Case Reports for the OSPAR List of Threatened and/or Declining Species and Habitats*. (2008).

OSPAR Commission. Intertidal Mytilus edulis beds on mixed and sandy sediments Quality Status Report 2010. *Case Reports for the OSPAR List of Threatened and/or Declining Species and Habitats*. (2010).

Zu Ermgassen, P. S. E. et al. The benefits of bivalve reef restoration: A global synthesis of underrepresented species. *Aquat. Conserv.* **30**, 2050–2065 (2020).

Zu Ermgassen, P. S. E. et al. European Native Oyster Reef Ecosystems Are Universally Collapsed. *Conservation Letters*. **e13068** (2024).

<u>Sabellaria reefs</u>

Achari, K. G. Polychaetes of the family Sabellariidae with special reference to their intertidal habitat. *Proc. Indian Natl. Sci. Acad.* **35**, 442–455 (1974).

Braithwaite, C. J. R., Robinson, R. J. & Jones, G. Sabellarids: a hidden danger or an aid to subsea pipelines? *Q. J. Eng. Geol. Hydrogeol.* **39**, 259–265; doi:10.1144/1470-9236/05-057 (2006).

Cunningham, P. N., Hawkins, S. J., Jones, H. D. & Burrows, M. T. The geographical distribution of Sabellaria alveolata (L.) in England, Wales and Scotland, with investigations into the community structure of and the effects of trampling on Sabellaria alveolata colonies. (Nature Conservancy Council, 1984).

Dias, A. S. & Paula, J. Associated fauna of Sabellaria alveolata colonies on the central coast of Portugal. *J. Mar. Biol. Assoc. U. K.* **81**, 169–170 (2001).

Dubois, S., Commito, J. A., Olivier, F. & Retière, C. Effects of epibionts on Sabellaria alveolata (L.) biogenic reefs and their associated fauna in the Bay of Mont Saint-Michel. *Estuar. Coast. Shelf Sci.* **68**, 635–646 (2006).

Foster-Smith, R. L. Sabellaria spinulosa reef in the Wash and North Norfolk Coast cSAC and its approaches: Part II, fine scale mapping of the spatial and temporal distribution of reefs and the development of techniques for monitoring condition (Natural England, 2004).

Hayward, P. & Ryland, J. *Handbook of the Marine Fauna of North-West Europe*. (Oxford University Press, 1998).

Hendrick, V. J. An appraisal of Sabellaria spinulosa reefs in relation to their management and conservation. PhD Thesis. (University of Newcastle Upon Tyne, 2007).

Holt, T. J., Rees, I. E., Hawkins, S. J. & Seed, R. Biogenic Reefs (Volume IX). An overview of dynamic and sensitivity characteristics for conservation management of marine SACs. (Scottish Association for Marine Science, 1998).

McIntosh, W. C. *A monograph of British Marine Annelids Volume IV, Part 1. Polychaeta - Hermellidae to Sabellariidae*. (The Ray Society London, 1922). doi:10.5962/bhl.title.54725

Pearce, B. The ecology of Sabellaria spinulosa reefs. Doctoral dissertation. (University of Plymouth, 2017).

Rees, H. L. et al. A comparison of benthic biodiversity in the North Sea, English Channel, and Celtic Seas. *ICES J. Mar. Sci.* **56**, 228–246; doi:10.1006/jmsc.1998.0438 (1999).

Reise, K. & Schubert, A. Macrobenthic turnover in the subtidal Wadden Sea - The Norderaue revisited after 60 years. *Helgol. Meeresunters.* **41**, 69–82; doi:10.1007/bf02365100 (1987).

Riesen, W. & Reise, K. Macrobenthos of the subtidal Wadden Sea - revisited after 55 years. *Helgol. Meeresunters.* **35**, 409–423; doi:10.1007/bf01999132 (1982).

Seiderer, L. J. & Newell, R. C. Analysis of the relationship between sediment composition and benthic community structure in coastal deposits: Implications for marine aggregate dredging. *ICES J. Mar. Sci.* **56**, 757–765; doi:10.1006/jmsc.1999.0495 (1999).

Smith, A. M., McGourty, C. R., Kregting L. and Elliot, A. Subtidal *Galeolaria hystrix* (Polychaeta: Serpulidae) reefs in Paterson Inlet, Stewart Island, New Zealand. *New Zealand Journal of Marine and Freshwater Research.* **39**(6), 1297-1304; doi:10.1080/00288330.2005.9517394 (2005).

Wilson, D. P. Sabellaria colonies at duckpool, North Cornwall, 1961–1970. *J. Mar. Biol. Assoc. U. K.* **51**, 509–580 (1971).

Supplementary References 2.

Figure 5 Reference list:

Seagrass

Fletcher, S., Saunders, J., Herbert, R., Roberts, C. & Dawson, K. 2012. Description of the ecosystem services provided by broad-scale habitats and features of conservation importance that are likely to be protected by marine protected areas in the marine conservation zone project area. Natural England commissioned reports, number 088.

Potts, T., Burdon, D., Jackson, E., Atkins, J., Saunders, J., Hastings, E. and Langmead, O., 2014. Do marine protected areas deliver flows of ecosystem services to support human welfare?. *Marine Policy*, **44,** pp.139-148.

Cordingley, A., Anderson, L., Matthews, S., Beach, M., Molloy, L., Whittaker, L., Van Rein, H., McNulty, J., Reeve, N., Parsons, J., Morgan, V., 2023. The universal Asset Service Matrix (uASM)

Orth, R., Harwell, M. and Inglis, G., 2006. Ecology of seagrass seeds and seagrass dispersal processes. *Seagrasses: Biology, ecology and conservation*, pp.111-133.

Simon, T.N. and Levitan, D.R., 2011. Measuring fertilization success of broadcast-spawning marine invertebrates within seagrass meadows. *The Biological Bulletin*, **220(1),** pp.32-38.

Lilley, R.J. and Unsworth, R.K., 2014. Atlantic Cod (Gadus morhua) benefits from the availability of seagrass (Zostera marina) nursery habitat. *Global Ecology and Conservation*, **2,** pp.367-377.

Irlandi, E.A., Orlando, B.A. and Ambrose Jr, W.G., 1999. Influence of seagrass habitat patch size on growth and survival of juvenile bay scallops, Argopecten irradians concentricus (Say). *Journal of Experimental Marine Biology and Ecology*, **235(1),** pp.21-43.

zu Ermgassen, P.S., DeAngelis, B., Gair, J.R., Ermgassen, S.Z., Baker, R., Daniels, A., MacDonald, T.C., Meckley, K., Powers, S., Ribera, M. and Rozas, L.P., 2021. Estimating and applying fish and invertebrate density and production enhancement from seagrass, salt marsh edge, and oyster reef nursery habitats in the Gulf of Mexico. *Estuaries and Coasts*, **44,** pp.1588-1603.

Garrard, S.L. and Beaumont, N.J., 2014. The effect of ocean acidification on carbon storage and sequestration in seagrass beds; a global and UK context. *Marine Pollution Bulletin*, **86(1-2),** pp.138-146.

Koweek, D.A., Zimmerman, R.C., Hewett, K.M., Gaylord, B., Giddings, S.N., Nickols, K.J., Ruesink, J.L., Stachowicz, J.J., Takeshita, Y. and Caldeira, K., 2018. Expected limits on the ocean acidification buffering potential of a temperate seagrass meadow. *Ecological Applications*, **28(7),** pp.1694-1714.

Saltmarsh

Fletcher, S., Saunders, J., Herbert, R., Roberts, C. & Dawson, K. 2012. Description of the ecosystem services provided by broad-scale habitats and features of conservation importance that are likely to be protected by marine protected areas in the marine conservation zone project area. Natural England commissioned reports, number 088.

Potts, T., Burdon, D., Jackson, E., Atkins, J., Saunders, J., Hastings, E. and Langmead, O., 2014. Do marine protected areas deliver flows of ecosystem services to support human welfare?. *Marine Policy*, **44,** pp.139-148.

Cordingley, A., Anderson, L., Matthews, S., Beach, M., Molloy, L., Whittaker, L., Van Rein, H., McNulty, J., Reeve, N., Parsons, J., Morgan, V., 2023. The universal Asset Service Matrix (uASM)

zu Ermgassen, P.S., DeAngelis, B., Gair, J.R., Ermgassen, S.Z., Baker, R., Daniels, A., MacDonald, T.C., Meckley, K., Powers, S., Ribera, M. and Rozas, L.P., 2021. Estimating and applying fish and invertebrate density and production enhancement from seagrass, salt marsh edge, and oyster reef nursery habitats in the Gulf of Mexico. *Estuaries and Coasts*, **44,** pp.1588-1603.

Wang, Z.A., Kroeger, K.D., Ganju, N.K., Gonneea, M.E. and Chu, S.N., 2016. Intertidal salt marshes as an important source of inorganic carbon to the coastal ocean. *Limnology and Oceanography*, **61(5),** pp.1916-1931.

Oysters

Fletcher, S., Saunders, J., Herbert, R., Roberts, C. & Dawson, K. 2012. Description of the ecosystem services provided by broad-scale habitats and features of conservation importance that are likely to be protected by marine protected areas in the marine conservation zone project area. Natural England commissioned reports, number 088.

Potts, T., Burdon, D., Jackson, E., Atkins, J., Saunders, J., Hastings, E. and Langmead, O., 2014. Do marine protected areas deliver flows of ecosystem services to support human welfare?. *Marine Policy*, **44,** pp.139-148.

Cordingley, A., Anderson, L., Matthews, S., Beach, M., Molloy, L., Whittaker, L., Van Rein, H., McNulty, J., Reeve, N., Parsons, J., Morgan, V., 2023. The universal Asset Service Matrix (uASM)

Scyphers, S.B., Powers, S.P., Heck Jr, K.L. and Byron, D., 2011. Oyster reefs as natural breakwaters mitigate shoreline loss and facilitate fisheries. *PloS one*, **6(8),** p.e22396

Lipcius, R.N., Zhang, Y., Zhou, J., Shaw, L.B. and Shi, J., 2021. Modeling oyster reef restoration: Larval supply and reef geometry jointly determine population resilience and performance. *Frontiers in Marine Science*, **8,** p.677640.

Theuerkauf, S.J., Burke, R.P. and Lipcius, R.N., 2015. Settlement, growth, and survival of eastern oysters on alternative reef substrates. *Journal of Shellfish Research*, **34(2),** pp.241-250.

zu Ermgassen, P.S., DeAngelis, B., Gair, J.R., Ermgassen, S.Z., Baker, R., Daniels, A., MacDonald, T.C., Meckley, K., Powers, S., Ribera, M. and Rozas, L.P., 2021. Estimating and applying fish and invertebrate density and production enhancement from seagrass, salt marsh edge, and oyster reef nursery habitats in the Gulf of Mexico. *Estuaries and Coasts*, **44,** pp.1588-1603.

Smyth, D., Roberts, D. The European oyster (Ostrea edulis) and its epibiotic succession. Hydrobiologia 655, 25–36 (2010). https://doi.org/10.1007/s10750-010-0401-x

Lemasson, A.J., Fletcher, S., Hall-Spencer, J.M. and Knights, A.M., 2017. Linking the biological impacts of ocean acidification on oysters to changes in ecosystem services: a review. *Journal of Experimental Marine Biology and Ecology*, **492,** pp.49-62.

Groner, M. L. *et al.* Oysters and eelgrass: potential partners in a high pCO2 ocean. *Ecology* **99**, 1802–1814 (2018).

Lee, H. Z. L., Davies, I. M., Baxter, J. M., Diele, K. & Sanderson, W. G. Missing the full story: First estimates of carbon deposition rates for the European flat oyster, Ostrea edulis. *Aquatic Conservation* **30**, 2076–2086 (2020).

de Paiva, J. N. S., Walles, B., Ysebaert, T. & Bouma, T. J. Understanding the conditionality of ecosystem services: The effect of tidal flat morphology and oyster reef characteristics on sediment stabilization by oyster reefs. *Ecological Engineering* **112**, 89–95 (2018).

Macroalgae

Mohring, M.B., Wernberg, T., Kendrick, G.A. and Rule, M.J., 2013. Reproductive synchrony in a habitat-forming kelp and its relationship with environmental conditions. *Marine biology*, **160,** pp.119-126.

Fredriksen, S. Food web studies in a Norwegian kelp forest based on stable isotope (δ13C and δ15N) analysis. *Marine Ecology. Progress Series* **260**, 71–81 (2003).

Steneck, R. S. *et al.* Kelp forest ecosystems: biodiversity, stability, resilience and future. *Environmental Conservation* **29**, 436–459 (2002).

Pfister, C. A., Altabet, M. A. & Weigel, B. L. Kelp beds and their local effects on seawater chemistry, productivity, and microbial communities. *Ecology* **100**, (2019).

Porzio, L., Buia, M.C. and Hall-Spencer, J.M., 2011. Effects of ocean acidification on macroalgal communities. *Journal of experimental marine biology and ecology*, **400(1-2),** pp.278-287.

Schlenger, A.J., Beas-Luna, R. and Ambrose, R.F., 2021. Forecasting ocean acidification impacts on kelp forest ecosystems. *PloS one*, **16(4),** p.e0236218.

Teagle, H., Hawkins, S.J., Moore, P.J., Smale, D.A., 2017. The role of kelp species as biogenic habitat formers in coastal marine ecosystems. Journal of Experimental Marine Biology and Ecology 492, 81–98. https://doi.org/10.1016/j.jembe.2017.01.017https://doi.org/10.1016/j.jembe.2017.01.017https://doi.org/10.1016/j.jembe.2017.01.017

Ortega, A., Geraldi, N., Alam, I., Kamau, A., Acinas, S., Logares, R., Gasol, J., Massana, R., Krause-Jensen, D., Duarte, C., 2019. Important contribution of macroalgae to oceanic carbon sequestration. Nature Geoscience 12. https://doi.org/10.1038/s41561-019-0421-8https://doi.org/10.1038/s41561-019-0421-8https://doi.org/10.1038/s41561-019-0421-8

Porzio, L., Buia, M.C. and Hall-Spencer, J.M., 2011. Effects of ocean acidification on macroalgal communities. Journal of experimental marine biology and ecology, 400(1-2), pp.278-287.

Schlenger, A.J., Beas-Luna, R. and Ambrose, R.F., 2021. Forecasting ocean acidification impacts on kelp forest ecosystems. PloS one, 16(4), p.e0236218.

Morris, R. L., Graham, T. D. J., Kelvin, J., Ghisalberti, M. & Swearer, S. E. Kelp beds as coastal protection: wave attenuation of Ecklonia radiata in a shallow coastal bay. *Annals of Botany* (2019) doi:10.1093/aob/mcz127.

Bertocci, I., Araújo, R., Oliveira, P. & Sousa-Pinto, I. REVIEW: Potential effects of kelp species on local fisheries. *Journal of Applied Ecology* **52**, 1216–1226 (2015).

Blamey, L. K. & Bolton, J. J. The economic value of South African kelp forests and temperate reefs: Past, present and future. *Journal of Marine Systems* **188**, 172–181 (2018).

Vásquez, JA, Zuñiga, S, Tala, F, Piaget, N, Rodríguez, DC, and Vega, JMA (2014a) Economic valuation of kelp forests in northern Chile: values of goods and services of the ecosystem. Journal of Applied Phycology, 26(2), 1081–1088. doi:10.1007/s10811-013-0173-6.

Seaweed Industries and Products in the UK: A Brief Review. In Sustainable Global Resources Of Seaweeds Volume 1, Vol. 1, , 249–263. doi:10.1007/978-3-030-91955-9_14.

Water quality maintenance: Jiang, Z, Liu, J, Li, S, and Chen, J (2020) Kelp cultivation effectively improves water quality and regulates phytoplankton community in a turbid, highly eutrophic bay. Science of the Total Environment, 707, 135561. doi:10.1016/j.scitotenv.2019.135561.

Bennett, S, Wernberg, T, Connell, SD, Hobday, AJ, Johnson, CR, and Poloczanska, ES (2016) The "Great Southern Reef": Social, ecological and economic value of Australia's neglected kelp forests. Marine and Freshwater Research, 67(1), 47–56. doi:10.1071/MF15232.

Eger, AM, Marzinelli, EM, Beas-luna, R, and Verges, A (2023) The value of ecosystem services in global marine kelp forests. Nature Communications, 14, 1894. doi:10.1038/s41467-023-37385-0.

Mudflat

Fletcher, S., Saunders, J., Herbert, R., Roberts, C. & Dawson, K. 2012. Description of the ecosystem services provided by broad-scale habitats and features of conservation importance that are likely to be protected by marine protected areas in the marine conservation zone project area. Natural England commissioned reports, number 088.

Potts, T., Burdon, D., Jackson, E., Atkins, J., Saunders, J., Hastings, E. and Langmead, O., 2014. Do marine protected areas deliver flows of ecosystem services to support human welfare?. *Marine Policy*, **44,** pp.139-148.

Cordingley, A., Anderson, L., Matthews, S., Beach, M., Molloy, L., Whittaker, L., Van Rein, H., McNulty, J., Reeve, N., Parsons, J., Morgan, V., 2023. The universal Asset Service Matrix (uASM)

Widdicombe, S., Spicer, J.I. and Kitidis, V., 2011. Effects of ocean acidification on sediment fauna. *Ocean acidification*, **9,** pp.176-191.

Watson, G.J., Murray, J.M., Schaefer, M. and Bonner, A., 2017. Bait worms: a valuable and important fishery with implications for fisheries and conservation management. *Fish and fisheries*, **18(2),** pp.374-388.

Maerl

Fletcher, S., Saunders, J., Herbert, R., Roberts, C. & Dawson, K. 2012. Description of the ecosystem services provided by broad-scale habitats and features of conservation importance that are likely to be protected by marine protected areas in the marine conservation zone project area. Natural England commissioned reports, number 088.

Potts, T., Burdon, D., Jackson, E., Atkins, J., Saunders, J., Hastings, E. and Langmead, O., 2014. Do marine protected areas deliver flows of ecosystem services to support human welfare?. *Marine Policy*, **44,** pp.139-148.

Cordingley, A., Anderson, L., Matthews, S., Beach, M., Molloy, L., Whittaker, L., Van Rein, H., McNulty, J., Reeve, N., Parsons, J., Morgan, V., 2023. The universal Asset Service Matrix (uASM)

Tuya, F. *et al.* Levelling-up rhodolith-bed science to address global-scale conservation challenges. *Science of the Total Environment* **892**, 164818 (2023).

Kamenos, N.A., Moore, P.G. and Hall-Spencer, J.M., 2004. Nursery-area function of maerl grounds for juvenile queen scallops Aequipecten opercularis and other invertebrates. *Marine Ecology Progress Series*, **274,** pp.183-189.

Hall-Spencer, J. M., Grall, J., Moore, P. G. & Atkinson, R. J. A. Bivalve fishing and maerl-bed conservation in France and the UK?retrospect and prospect. *Aquatic Conservation* **13**, S33–S41 (2003).

Riosmena-Rodríguez, R., Nelson, W. & Aguirre, J. *Rhodolith/Maërl Beds: A Global Perspective*. *Coastal research library* (2017). doi:10.1007/978-3-319-29315-8.

Van Der Heijden, L. H. & Kamenos, N. A. Reviews and syntheses: Calculating the global contribution of coralline algae to total carbon burial. *Biogeosciences* **12**, 6429–6441 (2015).

Mao, J. *et al.* Carbon burial over the last four millennia is regulated by both climatic and land use change. *Global Change Biology* **26**, 2496–2504 (2020).

Martin, S., 1 *et al. Community Metabolism in Temperate Maerl Beds. II. Nutrient Fluxes*. *MARINE ECOLOGY PROGRESS SERIES* vols. 335–335 31–41 https://www.int-res.com/articles/meps2007/335/m335p031.pdf (2007).

Legrand, E., Riera, P., Lutier, M., Coudret, J., Grall, J. and Martin, S., 2017. Species interactions can shift the response of a maerl bed community to ocean acidification and warming. *Biogeosciences*, **14(23),** pp.5359-5376.

Burdett, H., Perna, G., McKay, L., Broomhead, G. & Kamenos, N. Community-level sensitivity of a calcifying ecosystem to acute in situ CO2 enrichment. *Marine Ecology. Progress Series* **587**, 73–80 (2018).

Mussels

Folmer, Eelke O., Jan Drent, Karin Troost, Heike Büttger, Norbert Dankers, Jeroen Jansen, Marnix van Stralen, Gerald Millat, Marc Herlyn, and Catharina JM Philippart. "Large-scale spatial dynamics of intertidal mussel (Mytilus edulis L.) bed coverage in the German and Dutch Wadden Sea." Ecosystems 17 (2014): 550-566.

Kent, F.E., Gray, M.J., Last, K.S. and Sanderson, W.G., 2016. Horse mussel reef ecosystem services: evidence for a whelk nursery habitat supporting a shellfishery. International Journal of Biodiversity Science, *Ecosystem Services & Management*, **12(3),** pp.172-180.

Fitzer, S.C., Phoenix, V.R., Cusack, M. and Kamenos, N.A., 2014. Ocean acidification impacts mussel control on biomineralisation. *Scientific reports*, **4(1),** p.6218.
